# Unveiling the effects of interspecific competition: ecological consequences of competitive release after damming on *Salvelinus curilus* populations in a three-salmonid species coexistence system

**DOI:** 10.1007/s00442-026-05918-1

**Published:** 2026-06-26

**Authors:** Kenta Anraku, Akihiko Goto, Taihei Yamada, Kazutoshi Ueda, Kentaro Morita

**Affiliations:** 1 Graduate School of Agricultural and Life Sciences, Atmosphere and Ocean Research Institute, The University of Tokyo, Kashiwa, Chiba Japan; 2 Graduate School of Agricultural and Life Sciences, The University of Tokyo, Bunkyo, Tokyo Japan; 3 Atmosphere and Ocean Research Institute, The University of Tokyo, Kashiwa, Chiba Japan; 4 National Fisheries University, Fisheries Research and Education Agency, Shimonoseki, Yamaguchi Japan; 5 Faculty of Environmental Earth Science, Hokkaido University, Sapporo, Hokkaido Japan; 6 Public Consultant Co, Ltd, Sapporo, Hokkaido Japan; 7 Present Address: Nikko Field Station, Fisheries Technology Institute, Japan Fisheries Research and Education Agency, Nikko, Tochigi Japan

**Keywords:** Character displacement, Habitat fragmentation, Morphological adaptation, Niche partitioning, Stream ecology

## Abstract

Abiotic factors and biotic interactions, such as interspecific competition, are fundamental determinants of animal growth and survival. However, comprehensively and quantitatively assessing the impacts of interspecific competition while accounting for confounding abiotic factors remains a challenge in ecological research. We investigated changes in the ecological and morphological characteristics of southern Asian Dolly Varden (*Salvelinus curilus*, SADV) with competitive release in river systems fragmented by small dams. In sympatric reaches downstream from dams, where SADV coexisted with masu salmon (*Oncorhynchus masou*) and white-spotted charr (*Salvelinus leucomaenis*), these populations exhibited reduced growth, apparent survival, and body condition. The sympatric SADV developed subterminal jaws and smaller eyes, adaptations that allowed them to shift to benthic prey. Conversely, SADV in allopatric reaches upstream of dams exhibited increased population densities, enhanced growth rates, and generally improved body condition. Morphological traits, such as terminal jaws and enlarged eyes, reflected adaptations for enhanced foraging efficiency on terrestrial prey. These findings highlight the demographic, behavioral, and developmental effects of interspecific competition and competitive release on the morphology and ecology of these charr, providing valuable insights into how habitat fragmentation influences population dynamics and adaptive traits.

**Supplementary Information:**

The online version contains supplementary material available at https://doi.org/10.1007/s00442-026-05918-1.

## Introduction

Animal growth and survival are governed by interactions between abiotic environmental factors, such as temperature, and biotic factors, such as interspecific competition (MacArthur 1972). These factors rarely act independently; instead, their interactions are dynamic and complex, collectively influencing population structure and ecosystem processes. Condition-specific competition, whereby competitive interactions are shaped by abiotic factors, has substantial implications for the growth, survival, and distribution of animals (Dunson and Travis 1991; Taniguchi and Nakano 2000; Twomey et al. 2008). Disentangling the effects of abiotic and biotic factors, especially interspecific competition, on fitness components remains a considerable challenge because of the complex interplay between physical and biological processes.

In instances where the ecological niches of two sympatric species overlap, interspecific competition occurs for various resources, such as space and prey. Coexistence is often achieved through mechanisms of resource partitioning (Murray 1971; Aschehoug et al. 2016; Hiruma et al. 2023). For example, in sunfishes, the bluegill (*Lepomis macrochirus*), green sunfish (*L. cyanellus*), and pumpkinseed (*L. gibbosus*) primarily prey on vegetation dwellers when stocked allopatrically. However, when the three species coexist, the bluegill and pumpkinseed exhibit clear feeding niche shifts: bluegill primarily consume open-water zooplankton, whereas pumpkinseed shift to benthic in- and epifauna on the pond bottom. In contrast, the diet of the green sunfish remains largely unchanged as they continue to exploit vegetation-associated prey. These patterns suggest that such flexible niche shifts in sympatry facilitate stable coexistence among the three species (Werner and Hal 1976). In a field manipulation experiment conducted within an enclosure, the anole lizards (*Anolis gingivinus)* exhibited reduced growth rates in the presence of *A. wattsi*. Despite this impact, the two species successfully coexisted by partitioning their ecological niches. This niche differentiation was evident in variations in prey size and horizontal and vertical space utilization (Pacala and Roughgarden 1982). Mathematical modeling studies have theoretically demonstrated that interspecific competition influences both population dynamics and life histories (Nisbet and Gurney 2003). Nevertheless, for vertebrates with prolonged generation times spanning multiple years, comprehensively and quantitatively assessing the effects of being relieved from interspecific competition across generations in natural environments remains challenging. For example, it is difficult to maintain continuous rearing over successive generations in natural environments.

Stream-dwelling salmonids that defend their territories have widely been used as model species in natural ecosystems to evaluate the effects of interspecific competition on population dynamics. These territorial fish experience both intra- and interspecific competition (Nakano and Furukawa-Tanaka 1994; Fausch et al. 2021; Togaki et al. 2024). Their distribution within rivers is determined by complex interactions between abiotic factors (e.g., water temperature and river gradient), which vary with stream flow, and biotic factors (e.g., interspecific competition) (Dunson and Travis 1991; Fausch et al. 1994). In Japan, native stream-dwelling salmonids include the southern Asian Dolly Varden (*Salvelinus curilus*, SADV), white-spotted charr (*Salvelinus leucomaenis*, WSC), and masu salmon (*Oncorhynchus masou*, MS). The mechanisms of coexistence and the association between interspecific competition and morphological variation in these species have been extensively studied. For instance, in river reaches where all three species coexist, WSC and MS prey predominantly on terrestrial invertebrates, whereas SADV individuals consume benthic invertebrates (Morita et al. 2024). In reaches where only WSC and SADV are sympatric, SADV individuals shift their feeding behavior to target benthic prey (Nakano and Furukawa‐Tanaka 1994; Nakano et al. 1999; Fausch et al. 2021). Furthermore, SADV individuals develop shorter lower jaws adapted for benthic predation via character displacement (Nakano et al. 2020).

Small dams (e.g., check dams and weirs) disrupt river continuity (National Research Council 1996), impede the upstream movement of fish, and cause the disappearance and local extinctions of migratory species (Bergerot et al. 2013; Keijzer et al. 2024). Although large dams lead to considerable changes in abiotic environments, small dams primarily influence fish community dynamics without causing substantial differences in abiotic factors or invertebrate prey assemblages between upstream and downstream habitats (Brown et al. 2024). Instead of gradual changes along the river continuum, small dams cause abrupt shifts in fish species composition, leading to allopatric habitats for species with resident life histories. Comparing allopatric and sympatric populations in their natural state under similar habitat conditions offers a valuable opportunity to disentangle the effects of interspecific competition on populations from other abiotic environmental factors, such as river gradient and water temperature regimes.

The rivers examined in this study are inhabited by MS and WSC, whose populations are sustained by anadromous individuals, and SADV, which has a stream resident life history. The MS and WSC have become locally extinct upstream of the dams because of the migration barrier. Therefore, upstream reaches from the dams serve as allopatric habitats for SADV, whereas the downstream reaches and dam-free rivers form three-species sympatric reaches comprising MS, WSC, and SADV.

Experimental studies aimed at quantifying the effects of competitive release over multiple generations in vertebrates are scarce because of the challenges associated with conducting long-term studies. Consequently, the demographic and evolutionary processes underlying character displacement and character release remain largely unexplored. This study seeks to address this gap by investigating the effects of multi-generational long-term competitive release on SADV, focusing on their population density, body size, probability of maturing, stomach content composition, condition, and morphology. SADV generally lives for approximately 5–6 years (Shimoda et al. 1993); thus, a 30-year fragmentation period would, in simple calculation, span at least five or six generations.

We formulated several hypotheses regarding the effects of competitive release on SADV caused by dams. We predicted that allopatric SADV, free from interspecific competition and inhabiting the upstream reaches of dams, would (1) show increases in population densities; (2) exhibit enhanced growth rates and body condition, leading to earlier maturation; (3) increase consumption of terrestrial invertebrates, which are typically consumed by competing species; and (4) develop a terminal jaw morphology that resembles the morphology of competing species, which is suited for feeding on terrestrial invertebrates. We aimed to empirically evaluate the effects of competitive release over multiple generations on SADV resulting from long-term allopatric isolation due to dams in natural environments.

## Materials and methods

### Research site

**Fig. 1:**
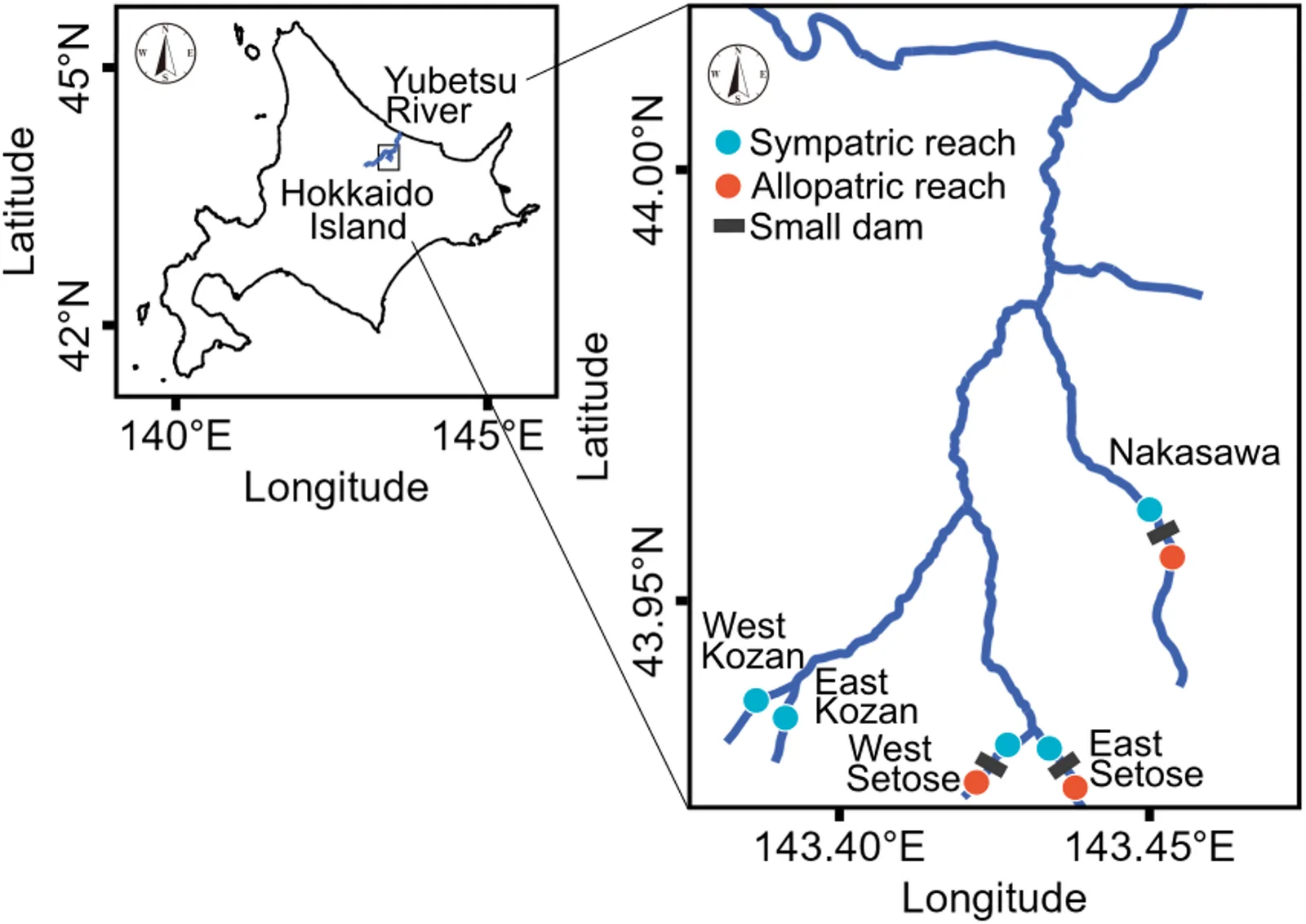
Study sites in the Setose River watershed of the Yubetsu River system, which flows into the Sea of Okhotsk. The survey was conducted in the upstream and downstream areas of the dams on the Nakasawa, East Setose, and West Setose Rivers, as well as in the undammed East Kozan and West Kozan Rivers. The allopatric study reaches of southern Asian Dolly Varden (SADV) are indicated by red circles (*n* = 3), and the sympatric study reaches (*n* = 5) are indicated by blue circles. Small dams are indicated by gray bars

A survey was conducted on five rivers in the Setose River watershed of the Yubetsu River system, flowing into the Sea of Okhotsk in Hokkaido, Japan (Fig. 1): the East Setose, West Setose, Nakasawa, East Kozan, and West Kozan Rivers. Three of the rivers, namely the East Setose, West Setose, and Nakasawa Rivers, featured erosion control weirs and small hydraulic facilities (hereafter referred to as dams) installed in 1994, 1995, and 1964, respectively. The small dam on the Nakasawa River functions as a water diversion weir. As the diversion gates were frequently opened until the early 1990s, water flowed through the open gates in the weir, allowing fish access upstream until 1995. After 1995, the diversion gate was closed, preventing upstream migration of fishes. Although these dams were small and did not retain water, their vertical drops, ranging from 2 to 5 m, effectively prevented the migration of anadromous MS and WSC.

In the three rivers where dams have been constructed (the East Setose, West Setose, and Nakasawa Rivers), 50 m study reaches were established at two locations upstream and downstream of the dams. In addition, a 50 m reach was established in each river without dams (the East Kozan and West Kozan Rivers). Changes in abiotic environmental conditions were detected within 50 m upstream and 10 m downstream of the dams; therefore, these areas were excluded from the study reaches. In these watersheds, some MS and WSC males exhibited a stream resident life history, whereas all MS and WSC females generally adopted a sea-run anadromous life history (Kato 1991; Yamamoto et al. 1999; Sahashi and Morita 2024). Therefore, the reproduction of MS and WSC is no longer possible upstream of the dam. Conversely, SADV individuals exhibit only a stream resident life history for both males and females (Shimoda et al. 1993; Fausch et al. 2024).

### Measurement of the riverine environment

In each 50 m study reach, the water width and river gradient were measured at 10 m intervals using a laser range finder (GLM 50 − 23 G, Robert Bosch Power Tools GmbH, Germany). Water depth was measured with a ruler, and velocity was quantified using a propeller-type current meter (Propeller-type Current Meter VR-301, KENEK Corp., Japan) at 1/3 and 2/3 of the stream width along transects perpendicular to the flow spaced at 10-m intervals. Gravel size (minor-axis diameter) was measured for randomly collected particles at seven places along the transects spaced at the same 10-m intervals. The sand was assigned a diameter of 1 mm diameter, whereas mud was assigned 0.05 mm diameter. Elevation and watershed areas were measured from the digital map of the Geospatial Information Authority of Japan (GSI). Water temperature loggers (HOBO TidbiT v2 Temperature Data Logger, Onset Computer Corp., USA) were installed in each reach, and the water temperature was recorded at hourly intervals (from 14-Jul to 31-Oct-2023).

### Fish collection

Fish were collected during the summer (foraging season) (14–17-Jul-2023) and autumn (breeding season for SADV) (26–31-Oct-2023), using two- or three-pass removal methods with electrofishing gear (300–400 V DC, Model 12-B, Smith-Root Inc., USA) at each 50 m study reach. The autumn survey period was likely conducted during the late breeding or post-breeding period for most individuals, as most dissected mature females (81%) lacked mature oocytes (fewer than 10 eggs) and were in a spent (post-spawning) condition. When the number of individuals captured in the two-pass samples did not decrease compared to that in the first-pass samples, three passes were conducted. The number of fish for each species in each study reach was estimated using the removal method (Model 2, mbh-Pollock, program CAPTURE, available at https://www.mbr-pwrc.usgs.gov/software/capture.shtml), and the population density (fish m^− 2^) was calculated. When the number of individuals used for stomach content analysis or fish samples was insufficient (less than 10 individuals) within the study reach, additional samples were obtained from upstream or downstream areas of the study reach.

### Fish measurement

Fish were anesthetized prior to measurement using an anesthetic (2-Phenoxyethanol, Wako Pure Chemical Industries, Ltd., Japan). Fork length and body weight of all salmonid fish were measured in the field. During the summer survey, stomach contents were collected through stomach pumping to flush out prey items by gastric lavage, which is highly effective (Saunders and Fausch 2012). Moreover, the lateral sides of approximately 10 individuals of each species in each reach were photographed. Stomach contents were fixed in 99% ethanol and frozen for preservation. After sampling, fish were released alive. During the autumn survey, random specimens of each species were photographed in the same manner as during the summer observations and then frozen and transported to the laboratory. These specimens were examined after thawing for sex, maturity (mature or immature), somatic mass (after removal of the gonads and organs), fork length, and body weight. Somatic mass was used as an index of potential post-reproductive energy reserves. Otoliths were collected, preserved in ethanol, and analyzed for age determination under a stereomicroscope (SZX10, Olympus Corp., Japan).

The scaled mass index (SMI) was calculated from the fork length, body weight, or somatic mass to evaluate the growth condition of individuals. The SMI (standardized weight corrected for mean length) was derived from the empirical correlation between weight and length (Peig and Green 2009). The SMI calculated using the total body weight was termed “body condition,” whereas that calculated using the somatic mass is referred to as the “somatic condition.” The somatic conditions were calculated for the autumn samples to assess the post-reproductive energy reserves.

### Benthos collection

Benthic invertebrates were collected from the riverbed to assess available benthic food resources within the study reaches. Samples were collected in July (14–17-Jul-2023). A total of 32 benthos samples were collected from four points (two pools and two riffles) within each study reach. Unfortunately, benthos samples from the West Kozan River were lost and could not be used in the analysis. Benthic invertebrates were sampled using Surber net samplers (25 × 25 cm, 0.5 mm mesh) and were immediately preserved in 99% ethanol for subsequent sorting.

### Collecting drift invertebrates

Drifting invertebrate prey were sampled at a single location in each river, except the West Kozan River, using a drift net (32 × 32 cm opening, 0.5 mm mesh) on the same day as the fish collection survey to assess drifting food resources within the study reaches. At each reach, a drift net was placed in the stream for 1–3.5 h starting at approximately 6:00 AM. The sieved water volume was estimated from the water velocity and depth at the center of the drift net. All drift samples were preserved in the same manner as the benthos samples.

### Stomach contents, benthos, and stream drift identification

The stomach contents, benthic invertebrates, and drifting invertebrates were sorted and identified at the order level. Terrestrial invertebrates were analyzed as a single category as in previous studies (Morita and Suzuki 1999; Nakano et al. 1999; Miyasaka et al. 2003). The number of individuals was counted, and their dry weight was measured for every order. Individual counts were based on the number of heads, and dry weights were measured to the nearest 0.001 g after drying at 60 °C for at least 48 h in a constant-temperature oven (OFW-600B, Asone Corp., Japan). Items that were too digested to identify or non-digestible, such as plants or stones, were excluded from the analysis.

### Data analysis

#### Definition of interspecific competition

Traditionally, interspecific competition is expressed by the following equation:

$$\frac{{dN}_{1}}{dt} = {r}_{1}{N}_{1} \left(1-\frac{{N}_{1}+{\alpha\:}_{12}{N}_{2}+{\alpha\:}_{13}{N}_{3}}{{K}_{1}}\right)$$

This extends the Lotka–Volterra competition model to encompass three species. Each coefficient is defined as follows: *K*_1_ is the carrying capacity of SADV; *r*_1_ is the intrinsic growth rate of SADV; *N*_1_ is the number of SADV; *N*_2_ is the number of MS; *N*_3_ is the number of WSC; _12_ is the competition coefficient from MS to SADV; and _13_ is the competition coefficient from WSC to SADV. The null hypothesis is _12_ = 0 and _13_ = 0. In this study, we assessed the presence of interspecific competition based on the condition that the competition coefficients _12_ and _13_ were greater than 0.

### River environment analyses

Multivariate analysis of variance (MANOVA) and generalized linear mixed models (GLMMs) were used to compare riverine environments between sympatric and allopatric reaches. Variables were natural-log transformed where necessary to improve normality. In the MANOVA, the response variables included water width, water depth, river gradient, natural-log-transformed water velocity, and natural-log-transformed gravel size. In the GLMMs, the response variables were the daily mean water temperature, natural-log-transformed dry weight of benthos, natural-log-transformed dry weight of aquatic invertebrates in stream drift (g m^− 3^), and natural-log-transformed dry weight of terrestrial invertebrates in stream drift (g m^− 3^). A small amount (0.0001 g m⁻³) was added to the terrestrial drift values before transformation to account for zero values. Competition status (sympatric = 0, allopatric = 1) was included as an explanatory variable in both analyses, and rivers were included as a random intercept in the GLMMs.

### Fish analysis

Ecological characteristics of sympatric SADV and allopatric SADV were compared using GLMMs. The response variables included the estimated number of fish, natural-log transformed fork length, body condition, somatic condition, maturity (immature = 0, mature = 1), and the proportion of terrestrial invertebrates in the diet. For the model with estimated number of fish as the response variable, the log-transformed area of the study reach was included as an offset term to compare fish density per unit area. Rivers were included as random intercepts in all models. Each model assessed the difference between competition status (categorical: sympatric = 0, allopatric = 1) in SADV through F-tests with a normal distribution or chi-square tests with Poisson or binomial distributions. The detailed models for each response variable are described in Table 1 and below. When a significant interaction between competition status and age was detected in the fork length analysis, we conducted Tukey’s post-hoc analyses examining differences between competition status within each age subgroup. When a significant interaction between competition status and maturity was detected in the somatic condition analysis, we conducted Tukey’s post-hoc analyses of the difference between competition statuses within each maturity group.

**Table 1:** Generalized linear mixed models (GLMMs) used to assess the influence of interspecific competition. The table summarizes the model structure for each response variable, together with the explanatory variables considered, including interaction terms, offset terms, and error distributions. The explanatory variables were competition status (sympatric or allopatric), season (summer or autumn), age (continuous), age group (0+, 1+, 2+, or 3+), fork length (continuous), sex (male or female), maturity (immature or mature), and species category (WSC, MS, allopatric SADV; sympatric SADV)

Response variable	Explanatory variables				Interaction term	Offset term	Distribution
Estimated number of SADV	Competition	Season			Competition × Season	Log (study reach area)	Poisson
Log (Fork length)	Competition	Age group (categorical)			Competition × Age group	None	Normal
Body condition	Competition	Season			Competition × Season	None	Normal
Somatic condition	Competition	Maturity	Age group (categorical)		Competition × Maturity	None	Normal
Probability of maturing	Competition	Fork length	Age (continuous)	Sex	None	None	Binomial
Proportion of terrestrial invertebrates in the stomach by number	Competition	Fork length			Competition × Fork length	None	Binomial
Lateral body morphology PCs (summer)	Competition	Fork length			Competition × Fork length	None	Normal
Head morphology PCs (autumn)	Competition	Fork length	Sex		Competition × Fork length Competition × Sex Fork length × Sex Competition × Fork length × Sex	None	Normal
Lateral body morphology PC1 (allopatric and sympatric SADV, MS, and WSC)	Species category	Fork length			Species category × Fork length	None	Normal

Preliminary analyses indicated that sex did not significantly affect size-at-age or somatic condition; therefore, sex and its interaction terms were excluded from the final analyses. Individuals aged 4 + and 5 + years were excluded from the analysis of fork length and conditions because of their small sample size (age 4+: *n* = 2; age 5+: *n* = 1). The interaction terms in the probability of maturing analysis were not significant, so these were excluded from the maturation reaction norm analysis.

### Geometric morphometric analysis

#### Comparison of allopatric and sympatric SADV morphology

**Fig. 2:**
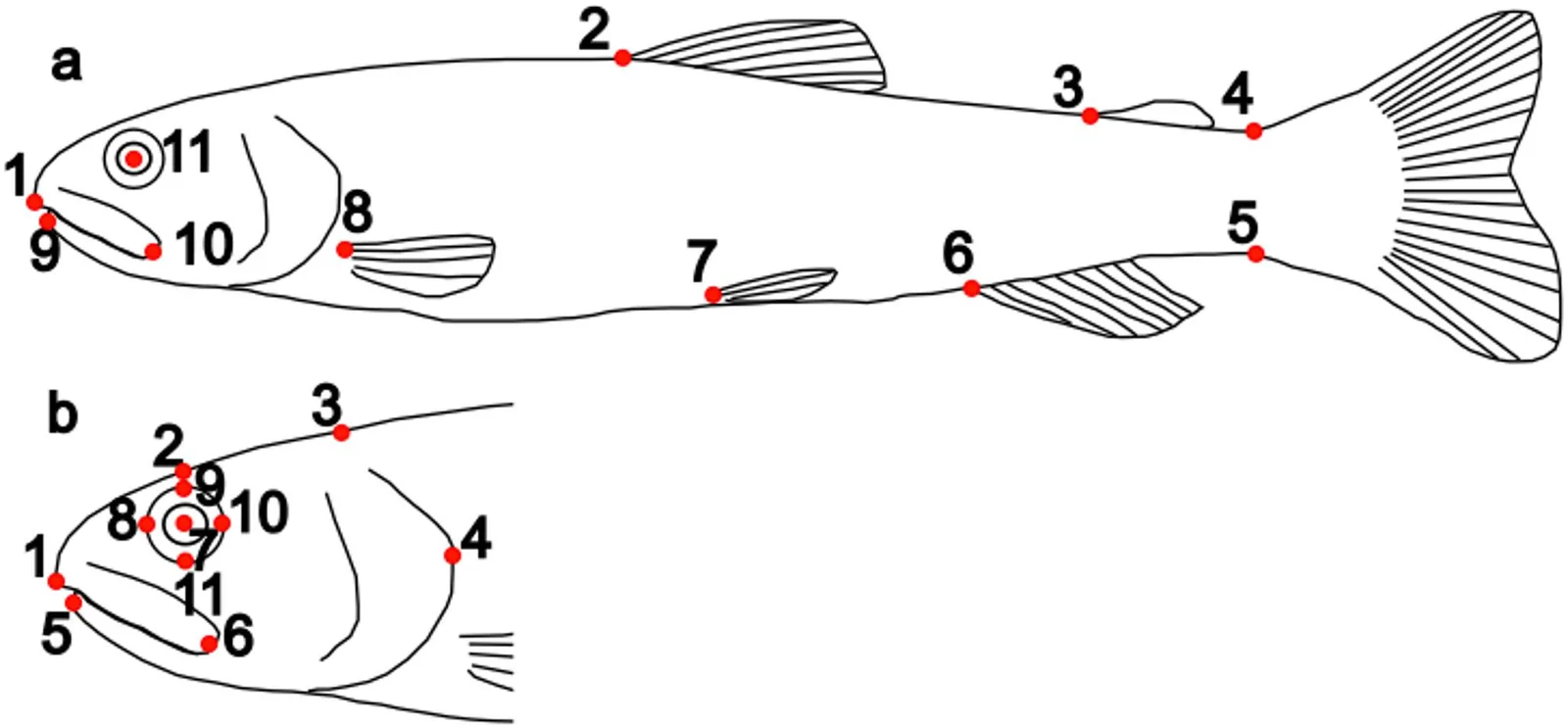
Placement of landmarks for the geometric morphometric analysis (red circles). (**a**) Eleven landmarks of lateral body shape; (**b**) 11 landmarks for head shape

We conducted a geometric morphometric analysis to examine the morphometric variation in allopatric and sympatric SADV. Landmark data were captured from photographs focusing on forms closely associated with skeletal features on the lateral body and head (Fig. 2). Morphometric software *tps-Util* (Ver. 1.78) and *tpsDig2* (Ver. 2.31) were used to obtain the landmark coordinates. There were 11 landmarks for the lateral body and head in the two-dimensional coordinates. Only individuals with fork lengths between 60 and 200 mm were included because those with fork lengths outside this range exhibited a morphology deviating from the normal shape.

Generalized Procrustes analysis (GPA) was performed on the coordinate data. The GPA separates the form of an organism into two components: centroid size and shape. This eliminates the non-shape variation caused by positioning, orientation, and scaling differences. Procrustes coordinates represent shape variables, with two assigned to each landmark in two-dimensional coordinates, whereas centroid size represents size, calculated as the square root of the sum of the squared distances of each landmark from its respective centroid (Zelditch et al. 2012). Procrustes ANOVA was applied to the GPA coordinate data to evaluate the morphological differences between allopatric and sympatric SADV, with centroid size (continuous) and competition status (categorical: sympatric = 0, allopatric = 1) as explanatory variables, with 9999 permutations performed. For the autumn data, sex (categorical: male = 0, female = 1) was included as an additional explanatory variable. Principal component analysis (PCA) was applied only to datasets for which competition status (the explanatory variable) showed significant morphological differences in the Procrustes ANOVA. In this study, PCA was not used for dimensionality reduction; rather, it was applied to orthogonalize the variables and examine their underlying structure (Jolliffe 2002). GLMMs were fitted for each PC (Table 1). The Bonferroni correction was applied to mitigate the multiple comparison problem across the PCs (18 tests for lateral body and head, respectively).

#### Comparison of the morphologies of the three salmonid species

We analyzed the morphometric variation among species categories, including allopatric SADV (*n* = 38), sympatric SADV (*n* = 55), MS (*n* = 43), and WSC (*n* = 46), using geometric morphometric analysis (GPA) based on lateral body views from the summer samples (Fig. 2a). Only individuals with fork lengths between 60 and 200 mm were included in this analysis. Eleven two-dimensional landmark coordinates were obtained and used for GPA (Fig. 2a). PCA was conducted on the GPA data to capture the variations in shape. The GLMMs were fitted using a normal distribution for PC1, which represented the primary axes of shape variation among species, and the rivers were included as random intercepts (Table 1).

All statistical analyses were conducted using R version 4.3.1 (R Core Team 2023). We used the *lme4* (Bates et al. 2015) and *glmmTMB* (Brooks et al. 2017) packages to fit GLMMs. *DHARMa* (Hartig 2024) was employed to check for overdispersion, while *geomorph* (Adams et al. 2024) was used for morphological analysis.

## Results

### Riverine environment

No significant differences in environmental variables were detected between allopatric and sympatric reaches in the MANOVA (*F*₅,₃₄ = 1.08, *p* = 0.391), which included water width, water depth, river gradient, water velocity, and gravel size. Similarly, GLMMs detected no significant differences in the mean daily water temperature, dry weight of benthic invertebrates, or dry weight of aquatic and terrestrial invertebrates in stream drift between the allopatric and sympatric reaches (Table 2, Table S1).

**Table 2:** Stream characteristics (mean ± SD) of the study reaches in the Setose River watershed of the Yubetsu River system, Hokkaido, Japan

Dam	Nakasawa		East Setose		West Setose		East Kozan	West Kozan
	Below	Above	Below	Above	Below	Above	Non	Non
Salmonids species	Sympatric	Allopatric	Sympatric	Allopatric	Sympatric	Allopatric	Sympatric	Sympatric
Water width (m)	2.2 ± 0.4	2.2 ± 0.6	3.0 ± 0.5	2.9 ± 0.6	2.6 ± 0.7	3.2 ± 0.9	2.0 ± 0.2	2.0 ± 0.5
Water depth (m)	0.14 ± 0.05	0.15 ± 0.04	0.17 ± 0.06	0.16 ± 0.08	0.14 ± 0.06	0.14 ± 0.06	0.13 ± 0.06	0.11 ± 0.03
Water velocity (m sec^− 1^)	0.45 ± 0.26	0.49 ± 0.31	0.56 ± 0.28	0.57 ± 0.32	0.48 ± 0.34	0.43 ± 0.18	0.29 ± 0.22	0.28 ± 0.16
Gravel size (mm)	40.1 ± 61.7	57.1 ± 122.2	76.3 ± 73.6	54.7 ± 60.0	86.5 ± 124.5	46.7 ± 73.4	64.5 ± 71.1	42.2 ± 47.9
Water temperature (℃)	10.5 ± 1.8	10.5 ± 1.7	12.5 ± 3.0	12.4 ± 2.9	11.4 ± 2.5	11.3 ± 2.5	9.7 ± 1.9	13.6 ± 3.4
River gradient (%)	5.4 ± 1.0	6.5 ± 2.3	5.0 ± 1.0	4.9 ± 1.3	6.7 ± 0.7	4.7 ± 1.4	9.8 ± 2.0	6.6 ± 2.1
Elevation (m)	323	328	342	348	343	349	345	349
Watershed area (km^2^)	2.46	2.41	4.21	3.61	3.40	3.35	1.24	1.92

### Population density

**Fig. 3:**
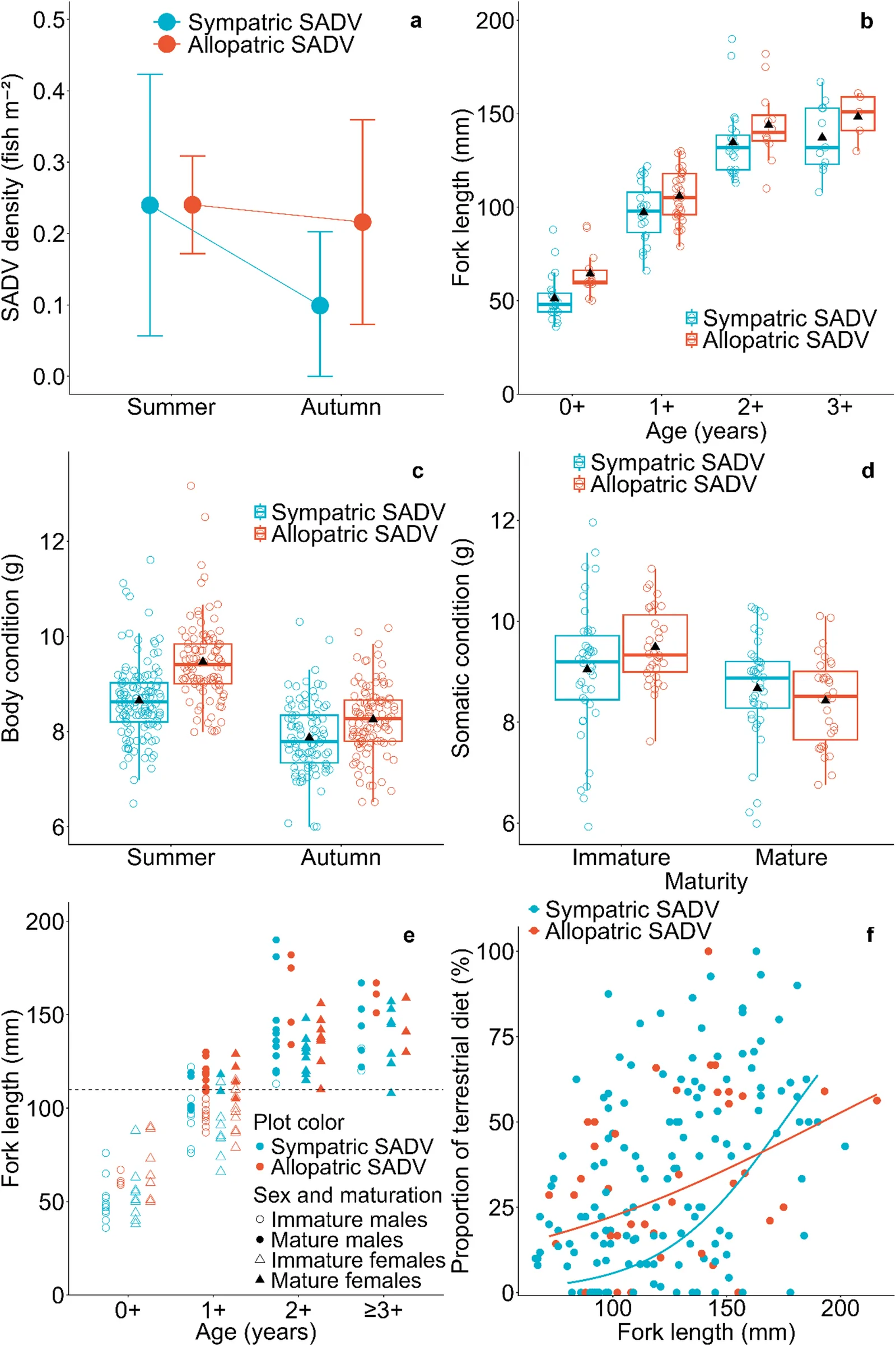
Ecological traits of southern Asian Dolly Varden (SADV) in sympatric and allopatric study reaches. (**a**) Changes in the population densities (mean ± SD) from summer to autumn. (**b**) Boxplots illustrating the fork length categorized by age group for allopatric (*n* = 64) and sympatric (*n* = 79) study reaches. The circles represent the actual measurements, and solid triangles represent the means. (**c**) Boxplots depicting the body condition (standardized body weight corrected for mean fork length) across allopatric (*n* = 205) and sympatric (*n* = 218) study reaches in summer and autumn. The circles represent individual measurements and the solid triangles represent the means. (**d**) Boxplots illustrating the somatic condition (scaled mass index calculated from somatic mass), categorized by maturity group for allopatric (*n* = 64) and sympatric (*n* = 79) study reaches. The circles represent the actual measurements, and the solid triangles represent the means. (**e**) Relationship between age, fork length, and maturity status (*n* = 147). The plots are categorized by maturity and competition statuses. For plotting, individuals aged 3 + and older were pooled and shown as “≥ 3+”. The dotted line indicates a 50% probability of maturation size as estimated from the GLMM. (**f**) Relationship between fork length and the numerical proportion of terrestrial invertebrates in the diet for allopatric (*n* = 39) and sympatric (*n* = 53) study reaches. Fitted logistic regression curves are shown for each competition status

In the summer and autumn surveys, we caught 82 and 79 allopatric SADV, 106 and 46 sympatric SADV, 113 and 72 WSC, and 715 and 284 individuals of MS, respectively (Fig. S1). During the summer months at this study site, the density of salmonids was substantially higher in sympatric reaches (total salmonids = 2.00 fish/m^2^, sympatric SADV = 0.24 fish/m^2^, MS = 1.47 fish/m^2^, WSC = 0.24 fish/m^2^) than in allopatric reaches (allopatric SADV = 0.24 fish/m^2^). In autumn, the densities of all three species declined; however, the overall salmonid densities remained higher in the sympatric reaches than in the allopatric reaches (total salmonids = 0.81 fish/m^2^, sympatric SADV = 0.10 fish/m^2^, MS = 0.55 fish/m^2^, WSC = 0.16 fish/m^2^, allopatric SADV = 0.22 fish/m^2^) (Fig. S2). The densities of all salmonids declined from summer to autumn, reflecting natural mortality losses over the summer after the recruitment of the next generation (age-0 + fish) in the spring. The decline in allopatric SADV was significantly less than that in sympatric groups (Table 3; Fig. 3a), suggesting lower apparent mortality rates in allopatric populations.

**Table 3:** Parameter estimates, test statistics, and *p*-values for each explanatory variable from generalized linear mixed models (GLMMs) for each response variable. The corresponding model structures are provided in Table 1. Categorical variables were coded as follows: competition status (sympatric = 0, allopatric = 1), season (summer = 0, autumn = 1), sex (male = 0, female = 1), and maturity (immature = 0, mature = 1). Age group (1+, 2+, or 3+) represents coefficients for each age class, with age 0 + as the reference category

Response variable	Explanatory variables	Coefficient	SE	Statistics	*p*-value
Number of SADV	(Intercept)	− 1.877	0.591	-	-
	Competition	0.127	0.290	χ^2^_1_ = 4.82	0.028
	Season	− 0.980	0.279	χ^2^_1_ = 8.33	0.004
	Competition × Season	0.837	0.410	χ^2^_1_ = 4.16	0.041
Log (Fork length)	(Intercept)	3.891	0.056	-	-
	Competition	0.207	0.048	*F*_1, 132_ = 131.6	< 0.001
	Age group			*F*_3, 133_ = 133.3	< 0.001
	Age-1+	0.661	0.042		
	Age-2+	0.989	0.042		
	Age-3+	1.068	0.053		
	Competition × Age group			*F*_3, 132_ = 132.2	0.013
	Age-1+	− 0.165	0.061		
	Age-2+	− 0.182	0.069		
	Age-3+	− 0.233	0.090		
Body condition	(Intercept)	8.656	0.070	-	-
	Competition	0.812	0.105	*F*_1, 276_ = 56.2	< 0.001
	Season	− 0.781	0.109	*F*_1, 417_ = 51.3	< 0.001
	Competition × Season	− 0.429	0.155	*F*_1, 417_ = 7.6	0.006
Somatic condition	(Intercept)	8.666	0.179	-	-
	Competition	0.279	0.225	*F*_1, 118_ = 1.4	0.232
	Maturity	− 0.412	0.307	*F*_1, 117_ = 1.6	0.199
	Age group			*F*_3, 130_ = 6.4	< 0.001
	Age-1+	0.930	0.223		
	Age-2+	0.370	0.336		
	Age-3+	0.400	0.364		
	Competition × Maturity	− 0.740	0.328	*F*_1, 136_ = 5.0	0.027
Probability of maturing	(Intercept)	− 19.253	4.201	-	-
	Fork length	0.173	0.039	χ^2^_1_ = 19.1	< 0.001
	Competition	− 0.109	0.780	χ^2^_1_ = 0.02	0.889
	Sex	0.681	0.713	χ^2^_1_ = 0.91	0.339
	Age	0.098	0.741	χ^2^_1_ = 0.02	0.894
Proportion of terrestrial invertebrates in the stomach	(Intercept)	− 6.556	0.897	-	-
	Fork length	0.037	0.006	χ^2^_1_ = 35.4	< 0.001
	Competition	3.958	1.217	χ^2^_1_ = 10.6	0.001
	Fork length × Competition	− 0.024	0.008	χ^2^_1_ = 7.3	0.006

### Growth

The SADV fork length was significantly influenced by the interaction between competition status and age (Table 3). In the post-hoc analyses conducted for each age class, allopatric SADV were significantly larger than sympatric SADV in age-0 + fish (*p* < 0.001), whereas no significant differences in fork length between competition status were detected in age-1+ (*p* = 0.290), age-2+ (*p* = 0.620), or age-3 + fish (*p* = 0.738) (Table 3). Despite experiencing higher conspecific densities, allopatric SADV tended to exhibit larger size-at-age than sympatric SADV, particularly among younger individuals (Fig. 3b). The significant interaction between competition status and age indicated that the difference in body size between sympatric and allopatric SADV decreased with increasing age.

Body condition (SMI derived from whole body weight) was significantly affected by competition status, season, and their interaction (Table 3). Allopatric SADV exhibited better body condition than sympatric SADV despite experiencing higher conspecific densities. Seasonal differences were evident, as body condition was better in summer than in autumn (during or at the end of the spawning season). However, the decrease in body condition from summer to autumn was more pronounced in allopatric SADV (Fig. 3c).

The somatic condition (SMI calculated from somatic mass) was significantly influenced by the interaction between competition status and maturity (Table 3). To further examine the significant interaction between competition status and maturity, we conducted post-hoc analyses within each maturity group. Allopatric SADV tended to have lower somatic condition than sympatric SADV in mature individuals (estimate = 0.462, *p* = 0.065). No significant difference in somatic conditions between competition statuses was detected in immature fish (estimate = − 0.279, *p* = 0.232). Although the difference was not statistically significant, the direction of the estimated effect in immature fish indicated a higher somatic condition in allopatric SADV than in sympatric SADV (Fig. 3d).

### Maturity

Only fork length significantly influenced the probability of maturing, whereas competition status, sex, and age did not significantly affect the probability of maturing (Table 3). These results suggested that fork length alone influenced the timing of maturation in SADV, which included both sexes, irrespective of competition status (Fig. 3e). The size at 50% probability of maturing, which included both sexes, was approximately 109.3 mm. The probability of maturing at 1 + years of age was 39.4% and 27.3% for allopatric and sympatric SADVs, respectively, with allopatric SADV individuals exhibiting a higher probability of maturing at younger ages because of their larger size at those ages.

### Stomach content analysis

**Fig. 4:**
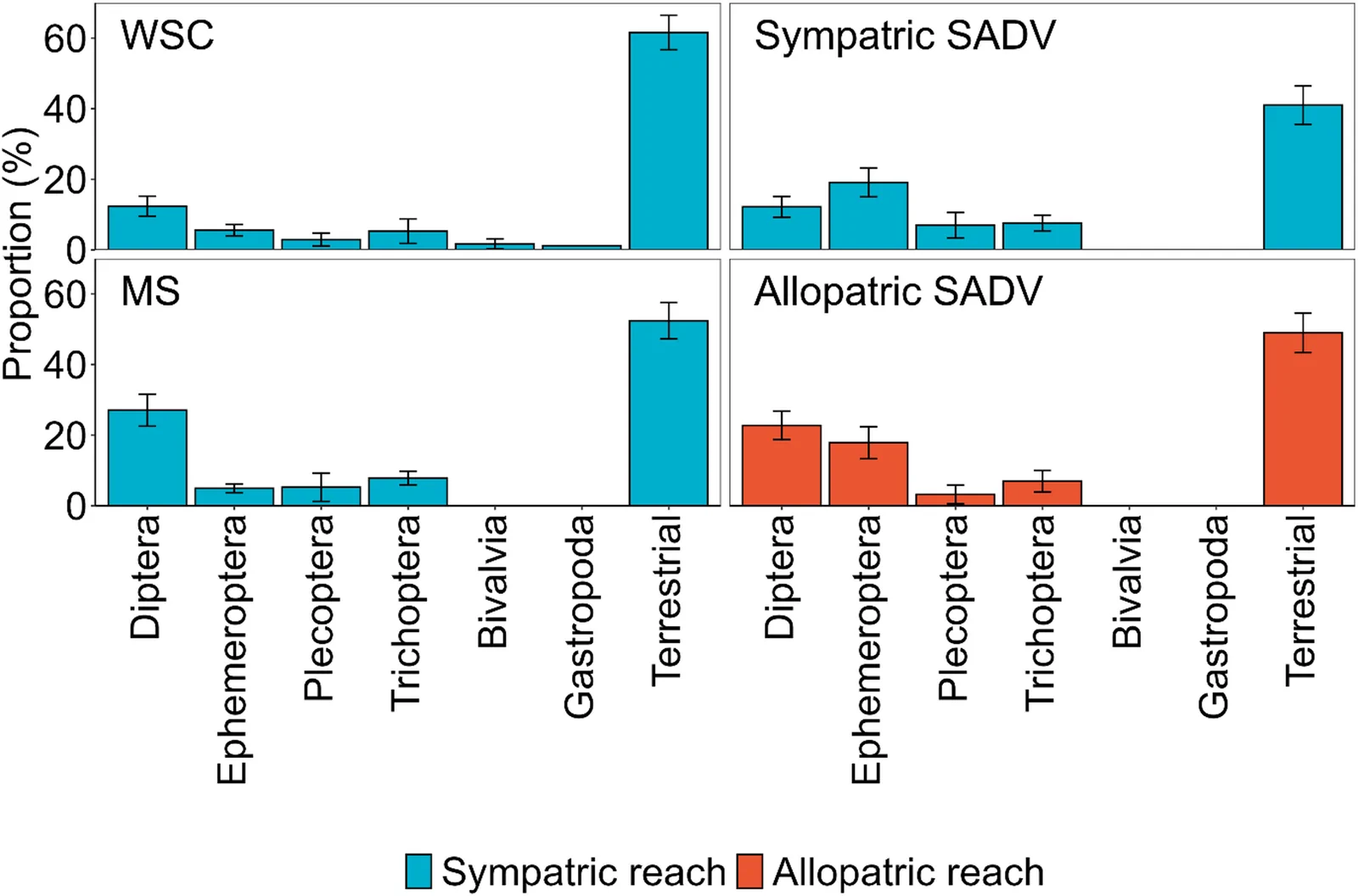
Diet compositions of white-spotted charr (WSC), masu salmon (MS), and southern Asian Dolly Varden (SADV) in the sympatric reaches and of SADV in allopatric reaches. Values are means ± SE based on dry weight (WSC, *n* = 47; MS, *n* = 43; sympatric SADV, *n* = 56; allopatric SADV, *n* = 38). Ephemeroptera, Plecoptera, Trichoptera, and Diptera indicate aquatic-stage larvae

Among the three salmonids, terrestrial invertebrates were the predominant prey and the most important resource (Fig. 4). The proportion of terrestrial invertebrate utilization by allopatric and sympatric SADVs was significantly influenced by competition status, fork length, and their interactions (Table 3). The proportion of terrestrial invertebrate utilization increased with fork length, and allopatric SADV demonstrated higher terrestrial invertebrate utilization than sympatric SADV, particularly in individuals with fork lengths < 160 mm (Fig. 3f).

### Geometric morphometric analysis

**Fig. 5:**
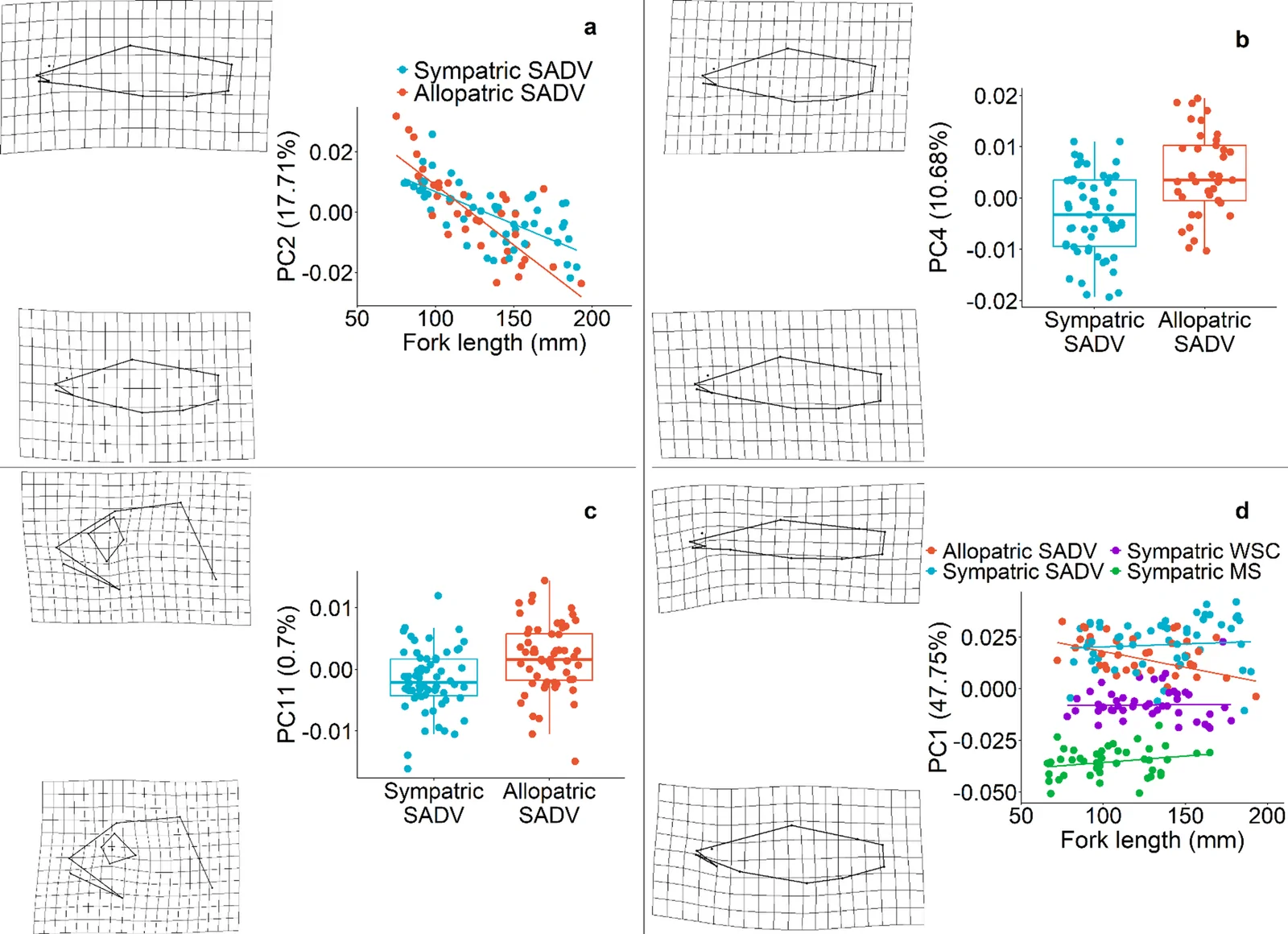
Morphological traits of southern Asian Dolly Varden (SADV) between sympatric and allopatric study reaches, with interspecific comparisons among stream salmonids in sympatric reaches. (**a**) Relationship between PC2 derived from the lateral body morphology and fork length of allopatric (*n* = 38) and sympatric (*n* = 55) SADV in summer, accompanied by fitted linear regressions for each competition status. (**b**) Boxplots illustrating the PC4 derived from the lateral body morphology of allopatric (*n* = 60) and sympatric (*n* = 62) SADV in autumn. (**c**) Boxplots depicting the PC11 derived from head morphology of allopatric (*n* = 60) and sympatric (*n* = 62) SADV in autumn. (**d**) Relationship between fork length and PC1 derived from the lateral body morphology of SADV in either allopatric (*n* = 38) or sympatric (*n* = 55) reaches, alongside white-spotted charr (WSC; *n* = 46) and masu salmon (MS; *n* = 43) in sympatric reaches in summer, with fitted linear regressions. Thin-plate spline graphs and wireframes featuring 11 marked landmarks illustrate morphological changes along each PC, transitioning from negative to positive ends. The dots in the boxplot represent each PC score

Procrustes ANOVA revealed that competition status significantly affected the lateral body morphology during summer (Table S2). GLMMs performed on PCs derived from lateral morphology in summer revealed a significant difference between competition statuses in PC4 and a significant interaction between competition status and fork length for PC2 (Table S3). Morphological differences between allopatric and sympatric SADVs along PC2 and PC4 primarily reflected variations in body height, whereas PC2 was also associated with differences in jaw morphology. Allopatric SADV demonstrated increased body height, elongated lower jaws, and developed terminal jaws as body length increased (Figs. 5a and b).

Procrustes ANOVA did not reveal significant differences between competition statuses in head morphology in summer (Table S2) or lateral body morphology in autumn (Table S2). However, significant differences in head morphology were detected between competition statuses in autumn (Table S2). GLMMs on PCs derived from head morphology in autumn showed a significant difference between competition status in PC11 (Table S3). Morphological changes along PC11 indicated larger eye sizes, more nasally positioned eyes, and elongated upper jaws in allopatric SADV in autumn (Figs. 5c and S3).

Regarding lateral morphology among the species categories, the GLMM for PC1 revealed significant differences among species categories, and a significant interaction between species categories and fork length (Table S4). Allopatric SADV exhibited morphological features resembling those of WSC and MS as fork length increased, with increased body height being a prominent feature of allopatric SADV (Fig. 5d).

## Discussion

### Effect of competition on population density, body size, body condition, and prey availability

Allopatric SADV indicated greater abundance than sympatric SADV, and their mortality rate from summer through autumn was apparently lower than that of sympatric SADV. The total salmonid density in the sympatric reaches was eight times higher than that in the allopatric reaches. These findings suggest that overcrowding of salmonids in sympatric reaches may have led to resource limitations, reduced growth rates, and increased mortality (Jonsson et al. 1998; Milner et al. 2003; Jonsson and Jonsson 2009).

The body size of allopatric SADV individuals was larger, and their body condition was better than that of sympatric SADV individuals. The dietary proportion of terrestrial invertebrates was greater in allopatric SADV, particularly among individuals smaller than 160 mm. Occupying profitable focal points and foraging on terrestrial invertebrates are crucial for growth and survival (Fausch 1984; Nislow et al. 1998). Competition for focal points in the pool is mainly influenced by body size, irrespective of species, with larger individuals occupying better focal points (Nakano and Furukawa-Tanaka 1994; Nakano 1995; Fausch et al. 2021). Several investigators reported that when WSC and SADV are sympatric, individual SADV typically switch to benthic foraging, especially as drifting invertebrate densities declined during summer (Nakano and Furukawa‐Tanaka 1994; Fausch et al. 1997, 2021; Nakano et al. 1999). Our results show that SADV in sympatry adopted this same benthic foraging strategy. In addition, SADV individuals are generally smaller than MS and WSC individuals during their juvenile stages (Ishigaki 1987) (Fig. S1). Such size asymmetry likely impedes their ability to establish profitable focal points in sympatric reaches, as even small differences in the body size of age-0 + fish affect dominance (Fausch and White 1986). Conversely, in allopatric reaches with low salmonid densities, SADV can select more profitable drift-feeding positions from an early age, as salmonids may exhibit a relatively wide foraging range from 0 + years of age (Ueno et al. 2009). An experimental study suggested that during summer, SADV experiences interspecific competition with WSC under water temperature conditions similar to those of the streams we studied, resulting in reduced growth rates (Watz et al. 2019). The high growth rate and body condition of SADV in allopatric populations may be attributed to their more frequent consumption of terrestrial invertebrates.

In populations experiencing competitive release, the relatively low densities of organisms occupying the same niche and reduced competition for food resources allow individuals to exploit more profitable niches at an earlier developmental stage and smaller body size (Rainville et al. 2021). This can lead to accelerated growth, enhanced physical condition, and improved survival rates (Kaspersson et al. 2012).

### Energy investment for reproduction

Unexpected results were obtained in the somatic condition analysis. We initially expected somatic conditions to be higher in allopatric SADV. This prediction was supported for immature individuals; however, in mature individuals, somatic conditions was lower in allopatric SADV, contrary to our expectation (Fig. 3d). The much higher body condition in allopatric SADV in summer and autumn (Fig. 3c), combined with the lower somatic condition in mature individuals, suggests greater reproductive output in the allopatric SADV (Fig. 3d), since the differences between body condition and somatic condition among mature fish may reflect differences in gonad weight and reproductive investment. SADV are iteroparous and do not die after reproduction. Iteroparous salmonids may increase their post-spawning survival by reducing egg size and number (Olofsson and Mosegaard 1999; Jonsson and Jonsson 1999; Kindsvater et al. 2016). Sympatric SADV may require sufficient energy reserves to survive after reproduction because of severe interspecific competition. In contrast, competitive release from interactions with MS and WSC may be associated with higher post-reproductive survival in allopatric SADV, potentially allowing greater allocation of energy to reproduction rather than somatic storage for survival.

### Maturation

The maturation onset of SADV was strongly influenced by fork length, regardless of competition status. In our study populations, SADV individuals typically matured at a fork length of approximately 110 mm (Fig. 3e). The threshold size at maturity observed in this study aligns with the trends observed in previous studies on SADV (Kitano and Nakano 1991; Sahashi and Morita 2018). The effect of body size on maturation timing is a common phenomenon among fish species, including other salmonids (Piché et al. 2008; Tsikliras and Stergiou 2014). The common threshold size at maturity and rapid initial growth of allopatric SADV would contribute to their earlier maturation. Allopatric SADV individuals had a higher maturation rate at 1 + years of age.

### Morphology

Some morphological differences are believed to arise from the energy availability of prey resources, whereas others may be attributed to interspecific competition or competitive release (Robinson and Wilson 1994). Interspecific competition drives character displacement, whereas competitive release promotes character release. Therefore, the observed morphological differences may have been influenced by interspecific competition for morphological traits. Species occupying overlapping niches may alter their morphology to enhance survival by shifting their niches in response to competition, whereas populations experiencing competitive release may adapt their morphology to optimize resource use and reproduction.

The SADV morphological changes in populations experiencing competitive release were observed in body height, jaw morphology, and eye size and position. Allopatric SADV exhibited greater body height. Increased body height can be attributed to improved body condition and greater access to high-quality food resources, such as terrestrial invertebrates. It may also provide resistance to downstream displacement during flooding (Yamada and Wada 2021). The increased body height observed in allopatric SADV resembled that of the competing species MS and WSC. This indicates that as body length increases, allopatric SADV exhibit morphological traits similar to those of MS and WSC, which are better adapted for swimming and exploiting drifting prey. A key finding is that allopatric SADV exhibit terminal and elongated jaws, and large eyes suitable for foraging on terrestrial invertebrates, which is consistent with the findings of Nakano et al. (2020). For example, the lake-dwelling benthic-foraging Dolly Varden (*Salvelinus malma*) has adapted to darker environments, resulting in smaller eyes, whereas individuals inhabiting the water column exhibit larger eyes (Esin et al. 2018). Furthermore, eye size correlates with the amount of energy available for eye development (Andersson et al. 2024), indicating that the large eyes of allopatric SADV may reflect their superior body condition.

Sympatric SADV exhibit a subterminal mouth well suited for foraging on benthic prey (Nakano et al. 2020). The results indicate that for the age-0 + SADV, which owing to their smaller body size are likely to be excluded from the more profitable foraging positions by age-0 + MS and WSC owing to their smaller body size, the simple act of picking benthic prey from the bed can feed back to result in the development of more subterminal mouths.

During the breeding season, salmonids show secondary sexual characteristics, notably a large forward extension of the jaw, particularly in males. The change in autumn head morphology indicated that allopatric SADV exhibited elongated upper jaw development and, contrary to intuition, a more anterior eye position. Competitive release may lead to a stronger expression of intraspecific competition in allopatric SADV and an enhanced expression of stronger secondary sexual traits. These secondary sexual traits may reflect a higher energy investment in reproduction.

## Conclusion

Allopatric SADV exhibited greater growth, body condition, and higher apparent survival than sympatric SADV. Furthermore, allopatric SADV exhibited a tendency to selectively feed on profitable terrestrial invertebrates and developed a terminal jaw and large eyes, which were suited to feeding on such invertebrates. Our results generally supported the hypotheses presented in the Introduction, although contrary to our prediction, allopatric mature SADV exhibited reduced somatic condition.

However, the population density of allopatric SADV did not approach the approximately eightfold higher salmonid densities observed in sympatric reaches. Moreover, sympatric SADV performance did not decline linearly in these high densities. This indicates that SADV can survive, grow, and coexist with other salmonids in sympatric reaches. All three salmonid species primarily depended on terrestrial invertebrates as their main prey resource. However, smaller individual SADV in sympatric populations increased the consumption of benthic invertebrates. Additionally, sympatric SADV displayed morphological adaptations to benthic foraging, such as subterminal jaws and smaller eyes. The observed changes may represent a mechanism to circumvent direct competition with MS and WSC, which actively target terrestrial invertebrates, through developmental pathways that alter morphology (Skúlason et al. 2019), and shift the SADV niche toward benthic foraging (Nakano et al. 2020).

Allopatric SADV, released from interspecific competition after damming, have managed to maintain relatively high population densities and exhibit superior ecological performance. This suggests that dam construction may provide limited long-term advantages for resident fish species capable of establishing populations upstream of the dam. However, the long-term effects of habitat fragmentation pose considerable challenges. Dam-induced fragmentation inhibits upstream movement, disrupts metapopulation structures (National Research Council 1996), and reduces population viability (Lande 1998). Long-term isolation increases extinction risk stemming from demographic, genetic, and environmental stochasticity (Morita et al. 2009, 2019; Tsuboi et al. 2013). Therefore, although the short-term positive benefits of dam construction may be evident for resident species, the long-term advantages are likely to be limited and require further investigation.

## Supplementary Information

Below is the link to the electronic supplementary material.


Supplementary Material 1


## Data Availability

The datasets used and/or analyzed in the current study are available from the corresponding author upon reasonable request.

## References

[CR1] Adams D, Collyer M, Kaliontzopoulou A, Baken E (2024) Geomorph: software for geometric morphometric analyses. R package version 4.0.8. https://cran.r-project.org/package=geomorph

[CR2] Andersson ML, Scharnweber K, Eklöv P (2024) Environmental and ecological drivers of eye size variation in a freshwater predator: a trade-off between foraging and predation risk. Funct Ecol 38:2470–2477. 10.1111/1365-2435.14655

[CR3] Aschehoug ET, Brooker R, Atwater DZ, Maron JL, Callaway RM (2016) The mechanisms and consequences of interspecific competition among plants. Annu Rev Ecol Evol Syst 47:263–281. 10.1146/annurev-ecolsys-121415-032123

[CR4] Bates D, Mächler M, Bolker B, Walker S (2015) Fitting linear mixed-effects models using lme4. J Stat Softw 67:1–48. 10.18637/jss.v067.i01

[CR5] Bergerot B, Hugueny B, Belliard J (2013) When local extinction and colonization of river fishes can be predicted by regional occupancy: the role of spatial scales. PLoS ONE 8:e84138. 10.1371/journal.pone.0084138 24367636 PMC3867478

[CR6] Brooks ME, Kristensen K, Benthem KJ, Magnusson A, Berg CW, Nielsen A, Skaug HJ, Mächler M, Bolker BM (2017) glmmTMB balances speed and flexibility among packages for zero-inflated generalized linear mixed modeling. R J 9:378–400. 10.32614/RJ-2017-066

[CR7] Brown RL, Charles D, Horwitz RJ, Pizzuto JE, Skalak K, Velinsky DJ, Hart DD (2024) Size-dependent effects of dams on river ecosystems and implications for dam removal outcomes. Ecol Appl 34:e3016. 10.1002/eap.3016 39138827

[CR8] Dunson WA, Travis J (1991) The role of abiotic factors in community organization. Am Nat 138:1067–1091. 10.1086/285270

[CR9] Esin EV, Markevich GN, Pichugin MY (2018) Juvenile divergence in adaptive traits among seven sympatric fish ecomorphs arises before moving to different lacustrine habitats. J Evol Biol 31:1018–1034. 10.1111/jeb.13283 29672982

[CR10] Fausch KD (1984) Profitable stream positions for salmonids: relating specific growth rate to net energy gain. Can J Zool 62:441–451. 10.1139/z84-067

[CR15] Fausch KD, White RJ (1986) Competition among juveniles of coho salmon, brook trout, and brown trout in a laboratory stream, and implications for Great Lakes tributaries. Trans Am Fish Soc 115:363–381. 10.1577/1548-8659(1986)115<363:CAJOCS>2.0.CO;2

[CR12] Fausch KD, Nakano S, Ishigaki K (1994) Distribution of two congeneric charrs in streams of Hokkaido Island, Japan: considering multiple factors across scales. Oecologia 100:1–12. 10.1007/BF00317124 28307021

[CR13] Fausch KD, Nakano S, Kitano S (1997) Experimentally induced foraging mode shift by sympatric charrs in a Japanese mountain stream. Behav Ecol 8:414–420. 10.1093/beheco/8.4.414

[CR14] Fausch KD, Nakano S, Kitano S, Kanno Y, Kim S (2021) Interspecific social dominance networks reveal mechanisms promoting coexistence in sympatric charr in Hokkaido, Japan. J Anim Ecol 90:515–527. 10.1111/1365-2656.13384 33159688

[CR11] Fausch KD, Morita K, Tsuboi J, Kanno Y, Yamamoto S, Kishi D, Dunham JB, Koizumi I, Hasegawa K, Inoue M, Sato T, Kitano S (2024) The past, present, and a future for native charr in Japan. Ichthyol Res 71:461–485. 10.1007/s10228-024-00955-3

[CR16] Hartig F (2024) Dharma: residual diagnostics for hierarchical (multi-level/mixed) regression models. R package version 0.4.7. https://CRAN.R-project.org/package=DHARMa

[CR17] Hiruma M, Takada H, Washida A, Koike S (2023) Dietary partitioning and competition between sika deer and Japanese serows in high elevation habitats. Mamm Res 68:305–315. 10.1007/s13364-023-00683-5

[CR18] Ishigaki K (1987) Studies on the biology in the early stages of two types of chars in Hokkaido. Res Bull Coll Exp Forests Hokkaido Univ 44:1121–1141

[CR19] Jolliffe IT (2002) Principal Components Analysis, 2nd Edition. Springer, New York

[CR21] Jonsson N, Jonsson B (1999) Trade-off between egg mass and egg number in brown trout. J Fish Biol 55:767–783. 10.1111/j.1095-8649.1999.tb00716.x

[CR20] Jonsson B, Jonsson N (2009) A review of the likely effects of climate change on anadromous Atlantic salmon *Salmo salar* and brown trout *Salmo trutta*, with particular reference to water temperature and flow. J Fish Biol 75:2381–2447. 10.1111/j.1095-8649.2009.02380.x 20738500

[CR22] Jonsson N, Jonsson B, Hansen LP (1998) The relative role of density-dependent and density‐independent survival in the life cycle of Atlantic salmon *Salmo salar*. J Anim Ecol 67:751–762. 10.1046/j.1365-2656.1998.00237.x

[CR23] Kaspersson R, Höjesjö J, Bohlin T (2012) Habitat exclusion and reduced growth: a field experiment on the effects of inter-cohort competition in young-of-the-year brown trout. Oecologia 169:733–742. 10.1007/s00442-012-2248-5 22271199

[CR24] Kato F (1991) Life histories of masu and amago salmon (*Oncorhynchus masou* and *Oncorhynchus rhodurus*). In: Groot C, Margolis L (eds) Pacific salmon life histories. UBC, Vancouver, pp 448–520

[CR25] Keijzer T, Barbarossa V, Marques A, Carvajal-Quintero JD, Huijbregts MAJ, Schipper AM (2024) Threats of dams to the persistence of the world’s freshwater fishes. Glob Change Biol 30:e17166. 10.1111/gcb.17166

[CR26] Kindsvater HK, Braun DC, Otto SP, Reynolds JD (2016) Costs of reproduction can explain the correlated evolution of semelparity and egg size: theory and a test with salmon. Ecol Lett 19:687–696. 10.1111/ele.12607 27146705

[CR27] Kitano S, Nakano S (1991) Growth, sexual maturity and food habit of the Dolly Varden charr (*Salvelinus malma*) in the Horobetsu Stream, Shiretoko Peninsula. Bol Shiretoko Mus 13:1–12 [in Japanese]

[CR28] Lande R (1998) Anthropogenic, ecological and genetic factors in extinction and conservation. Popul Ecol 40:259–269. 10.1007/BF02763457

[CR29] MacArthur RH (1972) Geographical ecology: patterns in the distribution of species. Princeton University Press, Princeton, NJ

[CR30] Milner NJ, Elliott JM, Armstrong JD, Gardiner R, Welton JS, Ladle M (2003) The natural control of salmon and trout populations in streams. Fish Res 62:111–125. 10.1016/S0165-7836(02)00157-1

[CR31] Miyasaka H, Nakano S, Furukawa-Tanaka T (2003) Food habit divergence between white-spotted charr and masu salmon in Japanese mountain streams: circumstantial evidence for competition. Limnology 4:1–10. 10.1007/s10201-002-0088-4

[CR34] Morita K, Suzuki T (1999) Shifts of food habit and jaw position of white-spotted charr after damming. J Fish Biol 55:1156–1162. 10.1111/j.1095-8649.1999.tb02066.x

[CR32] Morita K, Morita SH, Yamamoto S (2009) Effects of habitat fragmentation by damming on salmonid fishes: lessons from white-spotted charr in Japan. Ecol Res 24:711–722. 10.1007/s11284-008-0579-9

[CR33] Morita K, Sahashi G, Miya M, Kamada S, Kanbe T, Araki H (2019) Ongoing localized extinctions of stream-dwelling white-spotted charr populations in small dammed-off habitats of Hokkaido Island, Japan. Hydrobiologia 840:207–213. 10.1007/s10750-019-3891-1

[CR35] Morita K, Tsuboi J, Sahashi G, Futamura R, Ueda K, Kuroki M (2024) Longitudinal structuring of stream-fish assemblages: is niche partitioning observed in two-species systems applicable to three-species systems? Ichthyol Res 71:486–497. 10.1007/s10228-023-00937-x

[CR36] Murray BG (1971) The Ecological consequences of interspecific territorial behavior in birds. Ecology 52:414–423. 10.2307/1937624

[CR41] National Research Council (1996) Upstream: salmon and society in the Pacific Northwest. National Academies, Washington. 10.17226/4976

[CR37] Nakano S (1995) Competitive interactions for foraging microhabitats in a size-structured interspecific dominance hierarchy of two sympatric stream salmonids in a natural habitat. Can J Zool 73:1845–1854. 10.1139/z95-217

[CR40] Nakano S, Furukawa-Tanaka T (1994) Intra‐ and interspecific dominance hierarchies and variation in foraging tactics of two species of stream‐dwelling chars. Ecol Res 9:9–20. 10.1007/BF02347237

[CR38] Nakano S, Fausch KD, Kitano S (1999) Flexible niche partitioning via a foraging mode shift: a proposed mechanism for coexistence in stream-dwelling charrs. J Anim Ecol 68:1079–1092. 10.1046/j.1365-2656.1999.00355.x

[CR39] Nakano S, Fausch KD, Koizumi I, Kanno Y, Taniguchi Y, Kitano S, Miyake Y (2020) Evaluating a pattern of ecological character displacement: charr jaw morphology and diet diverge in sympatry versus allopatry across catchments in Hokkaido, Japan. Biol J Linn Soc 129:356–378. 10.1093/biolinnean/blz183

[CR42] Nisbet RM, Gurney W (2003) Modelling fluctuating population: reprint of first Edition (1982). Blackburn Press, Caldwell, NJ

[CR43] Nislow KH, Folt C, Seandel M (1998) Food and foraging behavior in relation to microhabitat use and survival of age-0 Atlantic salmon. Can J Fish Aquat Sci 55:116–127. 10.1139/f97-222

[CR44] Olofsson H, Mosegaard H (1999) Larger eggs in resident brown trout living in sympatry with anadromous brown trout. Ecol Freshw Fish 8:59–64. 10.1111/j.1600-0633.1999.tb00054.x

[CR45] Pacala S, Roughgarden J (1982) Resource partitioning and interspecific competition in two two-species insular Anolis lizard communities. Science 217:444–446. 10.1126/science.217.4558.444 17782979

[CR46] Peig J, Green AJ (2009) New perspectives for estimating body condition from mass/length data: the scaled mass index as an alternative method. Oikos 118:1883–1891. 10.1111/j.1600-0706.2009.17643.x

[CR47] Piché J, Hutchings JA, Blanchard W (2008) Genetic variation in threshold reaction norms for alternative reproductive tactics in male Atlantic salmon, *Salmo salar*. Proc Biol Sci 275:1571–1575. 10.1098/rspb.2008.0251 18426750 PMC2602666

[CR48] R Core Team (2023) R: A Language and Environment for Statistical Computing. R Foundation for Statistical Computing, Vienna, Austria. https://www.R-project.org/

[CR49] Rainville V, Filion A, Lussier I, Pépino M, Magnan P (2021) Does ecological release from distantly related species affect phenotypic divergence in brook charr? Oecologia 195:77–92. 10.1007/s00442-020-04822-6 33521849

[CR50] Robinson BW, Wilson DS (1994) Character release and displacement in fishes: a neglected literature. Am Nat 144:596–627. 10.1086/285696

[CR51] Sahashi G, Morita K (2018) Adoption of alternative migratory tactics: a view from the ultimate mechanism and threshold trait changes in a salmonid fish. Oikos 127:239–251. 10.1111/oik.03715

[CR52] Sahashi G, Morita K (2024) Partial migration in salmonids: focusing on Asian endemic masu salmon (*Oncorhynchus masou*) and white-spotted charr (*Salvelinus leucomaenis*). In: Lobon-Cervia J, Budy P, Gresswell R (eds) Advances in the ecology of stream-dwelling salmonids. Springer, Cham 44. 10.1007/978-3-031-44389-3_12

[CR53] Saunders WC, Fausch KD (2012) Grazing management influences the subsidy of terrestrial prey to trout in central Rocky Mountain streams (USA). Freshw Biol 57:1512–1529. 10.1111/j.1365-2427.2012.02804.x

[CR54] Shimoda K, Nakano S, Kitano S, Inoue M, Ono Y (1993) Present condition of stream fish assemblage in the Shiretoko Peninsula with special reference to human impacts. Hokkaido Univ Collect Sch Acad papers 6:17–27. https://hdl.handle.net/2115/37083

[CR55] Skúlason S, Parsons KJ, Svanbäck R, Räsänen K, Ferguson MM, Adams CE, Amundsen P-A, Bartels P, Bean CW, Boughman JW, Englund G, Guðbrandsson J, Hooker OE, Hudson AG, Kahilainen KK, Knudsen R, Kristjánsson BK, Leblanc CA-L, Jónsson Z, Öhlund G, Smith C, Snorrason SS (2019) A way forward with eco evo devo: an extended theory of resource polymorphism with postglacial fishes as model systems. Biol Rev Camb Philos Soc 94:1786–1808. 10.1111/brv.12534 31215138 PMC6852119

[CR56] Taniguchi Y, Nakano S (2000) Condition-specific competition: implications for the altitudinal distribution of stream fishes. Ecology 81:2027–2039. 10.1890/0012-9658(2000)081[2027:CSCIFT]2.0.CO;2

[CR57] Togaki D, Inoue M, Kawaguchi H, Yamamoto K (2024) Resource partitioning between non-native white-spotted charr and native red-spotted masu salmon in Shikoku, southwestern Japan: population- and individual-level analyses. Ichthyol Res 71:498–507. 10.1007/s10228-023-00941-1

[CR58] Tsikliras AC, Stergiou KI (2014) Size at maturity of Mediterranean marine fishes. Rev Fish Biol Fisheries 24:219–268. 10.1007/s11160-013-9330-x

[CR59] Tsuboi J, Iwata T, Morita K, Endou S, Oohama H, Kaji K (2013) Strategies for the conservation and management of isolated salmonid populations: lessons from Japanese streams. Freshw Biol 58:908–917. 10.1111/fwb.12096

[CR60] Twomey E, Morales V, Summers K (2008) Evaluating condition-specific and asymmetric competition in a species‐distribution context. Oikos 117:1175–1184. 10.1111/j.0030-1299.2008.16676.x

[CR61] Ueno T, Tanaka Y, Maruyama T (2009) Effects of adult white-spotted charr *Salvelinus leucomaenis* and masu salmon *Oncorhynchus masou masou* on focal points, distribution area and foraging frequency of both juveniles in a small tributary of Japanese mountain stream. Nippon Suisan Gakkaishi 75:802–809. 10.2331/suisan.75.802 [in Japanese]

[CR62] Watz J, Otsuki Y, Nagatsuka K, Hasegawa K, Koizumi I (2019) Temperature-dependent competition between juvenile salmonids in small streams. Freshw Biol 64:1534–1541. 10.1111/fwb.13325

[CR63] Werner EE, Hal DJ (1976) Niche shifts in sunfishes: experimental evidence and significance. Science 191:404–406. 10.1126/science.1246626 1246626

[CR64] Yamada H, Wada S (2021) Morphological evolution reduces downstream displacement in juvenile landlocked salmon. Evolution 75:1850–1861. 10.1111/evo.14281 34080690

[CR65] Yamamoto S, Morita K, Goto A (1999) Geographic variations in life-history characteristics of white-spotted charr (*Salvelinus leucomaenis*). Can J Zool 77:871–878. 10.1139/z99-055

[CR66] Zelditch M, Swiderski D, Sheets HD (2012) Geometric morphometrics for biologists: a primer, 2nd edn. Academic, London

