# Supplementary material for: Unveiling the effects of interspecific competition: ecological consequences of competitive release after damming on Salvelinus curilus populations in a three-salmonid species coexistence system
Source: Oecologia. 2026 Jun 26;208(7):87. doi: 10.1007/s00442-026-05918-1 (PMC13309472; doi:10.1007/s00442-026-05918-1)
Supplement: Supplementary file 1 — Supplementary Material 1 [file 442_2026_5918_MOESM1_ESM.docx]

**“Unveiling the effects of interspecific competition: Ecological consequences of competitive release after damming on *Salvelinus curilus* populations in a three-salmonid species coexistence system”**

Kenta Anraku*, Akihiko Goto, Taihei Yamada, Kazutoshi Ueda, Kentaro Morita

*Corresponding author: e-mail: [anraku-kenta818@g.ecc.u-tokyo.ac.jp](mailto:anraku-kenta818@g.ecc.u-tokyo.ac.jp)

**Table S1** Assessment of differences in river environments based on competition status (sympatric = 0, allopatric = 1) using generalized linear mixed models (GLMMs)

| Response variable | Explanatory  variables | Coefficient | SE | Statistics | p-value |
| --- | --- | --- | --- | --- | --- |
| Water temperature  (daily average) | (Intercept)  Competition | 11.531  - 0.125 | 0.686  0.197 | -  *F*_1, 838_ = 0.401 | -  0.527 |
| Dry weight of benthos | (Intercept)  Competition | 1.583  0.166 | 0.219  0.278 | -  *F*_1, 2_ = 0.308 | -  0.627 |
| Log (dry weight of aquatic invertebrates in stream drift) | (Intercept)  Competition | -9.126  -0.803 | 0.508  0.777 | -  *F*_1, 3_ = 0.936 | -  0.414 |
| Log (dry weight of terrestrial invertebrates in stream drift + 0.0001) | (Intercept)  Competition | -9.574  -3.198 | 1.573  2.403 | -  *F*_1, 3_ = 1.550 | -  0.313 |

**Table S2** Statistical analysis of the effects of centroid size (continuous), competition status (sympatric = 0, allopatric = 1), and sex (male = 0, female = 1) on head, side body, and ventral body morphology in southern Asian Dolly Varden using Procrustes ANOVA

| Season | Response variable | Explanatory variables | Sum of squares | Statistics | p-value |
| --- | --- | --- | --- | --- | --- |
| Summer | Head  morphology | Centroid size  Competition | 0.0901  0.0064 | *F*_1, 89_ = 15.000  *F*_1, 89_ = 1.064 | < 0.001  0.353 |
|  |  |  |  |  |  |
|  | Lateral body  morphology | Centroid size  Competition | 0.0088  0.0025 | *F*_1, 89_ = 13.702  *F*_1, 89_ = 3.809 | < 0.001  < 0.001 |
|  |  |  |  |  |  |
| Autumn | Head  morphology | Centroid size  Competition  Sex | 0.2089  0.0054  0.0240 | *F*_1, 118_ = 81.214  *F*_1, 118_ = 2.098  *F*_1, 118_ = 9.311 | < 0.001  0.045  < 0.001 |
|  |  |  |  |  |  |
|  | Lateral body  morphology | Centroid size  Competition  Sex | 0.0152  0.0009  0.0064 | *F*_1, 118_ = 34.207  *F*_1, 118_ = 2.029  *F*_1, 118_ = 5.243 | < 0.001  0.051  < 0.001 |

**Table S3** Analysis of the effects of competition status (sympatric = 0, allopatric = 1), season (summer = 0, autumn = 1), age (continuous), fork length (continuous), sex (male = 0, female = 1), and their interactions on the PCs from generalized Procrustes analysis of lateral, head, and ventral morphology in southern Asian Dolly Varden using GLMMs. Numbers in parentheses indicate the percentage of variance explained by each principal component. Explanatory variables and interaction terms were applied as needed. The analysis focused on the cases where competition was significant in Procrustes ANOVA

| Parts | Response variable | Explanatory variables | Coefficient | SE | Statistics | Bonferroni-adjusted  p-value |
| --- | --- | --- | --- | --- | --- | --- |
| Summer |  |  |  |  |  |  |
|  |  |  |  |  |  |  |
| Lateral morphology | PC1  (21.9 %) | (Intercept)  Competition  Fork length  Competition ×  Fork length | -0.0191  0.0123  0.0001  -0.0001 | 0.0071  0.0104  4.84E-05  0.0001 | -  *F*_1, 88_ = 2.519  *F*_1, 88_ = 6.311  *F*_1, 86_ = 2.541 | -  1.000  0.249  1.000 |
|  |  |  |  |  |  |  |
|  | PC2  (17.7 %) | (Intercept)  Competition  Fork length  Competition ×  Fork length | 0.0284  0.0204  -0.0002  -0.0002 | 0.0048  0.0073  3.39E-05  0.0001 | -  *F*_1, 87_ = 3.342  *F*_1, 80_ = 81.826  *F*_1, 87_ = 10.665 | -  1.000  < 0.001  0.028 |
|  |  |  |  |  |  |  |
|  | PC3  (12.7 %) | (Intercept)  Competition  Fork length  Competition ×  Fork length | 0.0115  -0.006  -0.0001  0.0001 | 0.0057  0.0086  4.00E-05  0.0001 | *-*  *F*_1, 87_ = 2.004  *F*_1, 82_ = 4.254  *F*_1, 87_ = 1.117 | -  1.000  0.762  1.000 |
|  |  |  |  |  |  |  |
|  | PC4  (10.7 %) | (Intercept)  Competition  Fork length  Competition ×  Fork length | -0.0031  -0.0021  -6.07E-07  0.0001 | 0.005  0.0071  3.33E-05  0.0001 | *-*  *F*_1, 88_ = 13.541  *F*_1, 88_ = 0.515  *F*_1, 85_ = 1.468 | -  0.007  1.000  1.000 |
|  |  |  |  |  |  |  |
|  | PC5  (8.0 %) | (Intercept)  Competition  Fork length  Competition ×  Fork length | 0.0051  -0.0084  -3.78E-05  0.0001 | 0.0046  0.0069  3.22E-05  0.0001 | *-*  *F*_1, 88_ = 0.036  *F*_1, 87_ = 0.384  *F*_1, 86_ = 1.399 | -  1.000  1.000  1.000 |
|  |  |  |  |  |  |  |
|  | PC6  (6.0 %) | (Intercept)  Competition  Fork length  Competition ×  Fork length | -0.0064  0.0113  3.99E-05  -0.0001 | 0.0039  0.0061  2.80E-05  4.59E-05 | *-*  *F*_1, 86_ = 4.646  *F*_1, 73_ = 0.611  *F*_1, 87_ = 1.812 | -  0.610  1.000  1.000 |
|  |  |  |  |  |  |  |
|  | PC7  (5.8 %) | (Intercept)  Competition  Fork length  Competition ×  Fork length | 0.0006  -0.0021  -9.98E-07  6.55E-06 | 0.0038  0.0062  2.75E-05  4.69E-05 | *-*  *F*_1, 79_ = 0.693  *F*_1, 24_ = 0.001  *F*_1, 87_ = 0.018 | -  1.000  1.000  1.000 |
|  |  |  |  |  |  |  |
|  | PC8  (5.2 %) | (Intercept)  Competition  Fork length  Competition ×  Fork length | -0.0051  0.0018  4.40E-05  -2.61E-05 | 0.0035  0.0057  2.53E-05  4.32E-05 | *-*  *F*_1, 79_ = 1.374  *F*_1, 24_ = 2.097  *F*_1, 87_ = 0.336 | -  1.000  1.000  1.000 |
|  |  |  |  |  |  |  |
|  | PC9  (3.6 %) | (Intercept)  Competition  Fork length  Competition ×  Fork length | 0.0029  -0.0079  -1.25E-05  3.78E-05 | 0.0029  0.0047  2.08E-05  3.55E-05 | *-*  *F*_1, 79_ = 7.061  *F*_1, 24_ = 0.005  *F*_1, 87_ = 1.04 | -  0.172  1.000  1.000 |
|  |  |  |  |  |  |  |
|  | PC10  (2.6 %) | (Intercept)  Competition  Fork length  Competition ×  Fork length | -0.001  0.004  4.31E-06  -2.27E-05 | 0.0026  0.0041  1.86E-05  3.10E-05 | *-*  *F*_1, 83_ = 1.15  *F*_1, 48_ = 0.024  *F*_1, 88_ = 0.509 | -  1.000  1.000  1.000 |
|  |  |  |  |  |  |  |
|  | PC11  (2.1 %) | (Intercept)  Competition  Fork length  Competition ×  Fork length | 0.0006  -0.0056  -2.10E-06  4.26E-05 | 0.0024  0.0036  1.67E-05  2.72E-05 | *-*  *F*_1, 87_ = 0.016  *F*_1, 83_ = 0.618  *F*_1, 87_ = 2.407 | -  1.000  1.000  1.000 |
|  |  |  |  |  |  |  |
|  | PC12  (1.1 %) | (Intercept)  Competition  Fork length  Competition ×  Fork length | -0.0016  -0.0011  9.66E-06  1.45E-05 | 0.0017  0.0026  1.20E-05  1.98E-05 | *-*  *F*_1, 85_ = 1.435  *F*_1, 62_ = 1.667  *F*_1, 88_ = 0.514 | -  1.000  1.000  1.000 |
|  |  |  |  |  |  |  |
|  | PC13  (0.8 %) | (Intercept)  Competition  Fork length  Competition ×  Fork length | 0.0001  0.0013  -3.62E-09  -1.14E-05 | 0.0014  0.0022  1.01E-05  1.70E-05 | *-*  *F*_1, 82_ = 0.058  *F*_1, 43_ = 0.134  *F*_1, 88_ = 0.428 | -  1.000  1.000  1.000 |
|  |  |  |  |  |  |  |
|  | PC14  (0.7 %) | (Intercept)  Competition  Fork length  Competition ×  Fork length | 0.0029  -0.0048  -1.97E-05  3.15E-05 | 0.0013  0.002  9.10E-06  1.55E-05 | *-*  *F*_1, 79_ = 2.18  *F*_1, 24_ = 1.343  *F*_1, 87_ = 3.775 | -  1.000  1.000  0.995 |
|  |  |  |  |  |  |  |
|  | PC15  (0.5 %) | (Intercept)  Competition  Fork length  Competition ×  Fork length | -0.0009  0.001  6.72E-06  -6.91E-06 | 0.0011  0.0018  7.81E-06  1.33E-05 | *-*  *F*_1, 79_ = 0.092  *F*_1, 24_ = 0.366  *F*_1, 87_ = 0.247 | -  1.000  1.000  1.000 |
|  |  |  |  |  |  |  |
|  | PC16  (0.4 %) | (Intercept)  Competition  Fork length  Competition ×  Fork length | 0.0002  0.0006  -1.45E-06  -5.08E-06 | 0.001  0.0016  7.28E-06  1.24E-05 | *-*  *F*_1, 79_ = 0.065  *F*_1, 24_ = 0.165  *F*_1, 87_ = 0.153 | -  1.000  1.000  1.000 |
|  |  |  |  |  |  |  |
|  | PC17  (0.3 %) | (Intercept)  Competition  Fork length  Competition ×  Fork length | 0.0008  -0.0008  -6.97E-06  7.66E-06 | 0.0009  0.0015  6.62E-06  1.13E-05 | *-*  *F*_1, 79_ = 0.335  *F*_1, 24_ = 0.517  *F*_1, 87_ = 0.421 | -  1.000  1.000  1.000 |
|  |  |  |  |  |  |  |
|  | PC18  (0.2 %) | (Intercept)  Competition  Fork length  Competition ×  Fork length | -0.0009  0.0013  7.71E-06  -1.17E-05 | 0.0007  0.0011  5.12E-06  8.59E-06 | *-*  *F*_1, 82_ = 0.733  *F*_1, 41_ = 0.715  *F*_1, 88_ = 1.747 | -  1.000  1.000  1.000 |
|  |  |  |  |  |  |  |
|  |  |  |  |  |  |  |
| Autumn |  |  |  |  |  |  |
|  |  |  |  |  |  |  |
| Head  morphology | PC1  (55.0 %) | (Intercept)  Competition  Fork length  Sex  Competition ×  Fork length  Competition ×  Sex  Fork length ×  Sex  Competition ×  Fork length ×  Sex | 0.1594  0.0333  -0.0015  -0.0684  -0.0004  -0.0174  0.0009  0.0002 | 0.0171  0.0227  0.0001  0.0259  0.0002  0.0351  0.0002  0.0003 | *-*  *F*_1, 72_ = 1.086  *F*_1, 112_ = 313.729  *F*_1, 113_ = 76.298  *F*_1, 114_ = 3.328  *F*_1, 113_ = 0.875  *F*_1, 111_ = 44.964  *F*_1, 111_ = 0.55 | -  1.000  < 0.001  < 0.001  1.000  1.000  < 0.001  1.000 |
|  |  |  |  |  |  |  |
|  | PC2  (10.5 %) | (Intercept)  Competition  Fork length  Sex  Competition ×  Fork length  Competition ×  Sex  Fork length ×  Sex  Competition ×  Fork length ×  Sex | 0.036  -0.0505  -0.0003  -0.0254  0.0005  0.0483  0.0002  -0.0004 | 0.0156  0.0201  0.0001  0.0225  0.0002  0.0306  0.0002  0.0003 | *-*  *F*_1, 111_ = 2.121  *F*_1, 111_ = 1.616  *F*_1, 112_ = 2.967  *F*_1, 111_ = 6.006  *F*_1, 112_ = 0.092  *F*_1, 110_ = 0.209  *F*_1, 110_ = 2.905 | -  1.000  1.000  1.000  0.285  1.000  1.000  1.000 |
|  |  |  |  |  |  |  |
|  | PC3  (9.1 %) | (Intercept)  Competition  Fork length  Sex  Competition ×  Fork length  Competition ×  Sex  Fork length ×  Sex  Competition ×  Fork length ×  Sex | -0.0228  -0.0059  0.0002  0.0138  6.41E-05  -0.0029  -6.80E-05  2.99E-05 | 0.0147  0.0192  0.0001  0.0215  0.0002  0.0292  0.0002  0.0002 | *-*  *F*_1, 107_ = 0.278  *F*_1, 111_ = 8.687  *F*_1, 112_ = 3.032  *F*_1, 112_ = 0.408  *F*_1, 113_ = 0.008  *F*_1, 110_ = 0.192  *F*_1, 110_ = 0.016 | -  1.000  0.007  1.000  1.000  1.000  1.000  1.000 |
|  |  |  |  |  |  |  |
|  | PC4  (7.5 %) | (Intercept)  Competition  Fork length  Sex  Competition ×  Fork length  Competition ×  Sex  Fork length ×  Sex  Competition ×  Fork length ×  Sex | -0.002  0.0052  4.24E-05  0.0113  -0.0001  -0.0301  -0.0001  0.0003 | 0.0139  0.0182  0.0001  0.0205  0.0001  0.0278  0.0002  0.0002 | *-*  *F*_1, 103_ = 2.36  *F*_1, 111_ = 0.015  *F*_1, 112_ = 0.039  *F*_1, 112_ = 0.003  *F*_1, 113_ = 1.673  *F*_1, 110_ = 0.178  *F*_1, 111_ = 2.059 | -  1.000  1.000  1.000  1.000  1.000  1.000  1.000 |
|  |  |  |  |  |  |  |
|  | PC5  (4.0 %) | (Intercept)  Competition  Fork length  Sex  Competition ×  Fork length  Competition ×  Sex  Fork length ×  Sex  Competition ×  Fork length ×  Sex | 0.0026  0.0047  -1.69E-05  -0.0018  -4.43E-05  -0.0061  1.47E-05  3.96E-05 | 0.0103  0.0137  0.0001  0.0156  0.0001  0.0212  0.0001  0.0002 | *-*  *F*_1, 72_ = 0.253  *F*_1, 112_ = 0.38  *F*_1, 113_ = 0.109  *F*_1, 114_ = 0.105  *F*_1, 113_ = 0.077  *F*_1, 111_ = 0.164  *F*_1, 111_ = 0.052 | -  1.000  1.000  1.000  1.000  1.000  1.000  1.000 |
|  |  |  |  |  |  |  |
|  | PC6  (3.7 %) | (Intercept)  Competition  Fork length  Sex  Competition ×  Fork length  Competition ×  Sex  Fork length ×  Sex  Competition ×  Fork length ×  Sex | 0.0061  -0.0166  -2.25E-05  0.0071  0.0001  0.002  -0.0001  -6.28E-06 | 0.0094  0.0125  0.0001  0.0141  0.0001  0.0191  0.0001  0.0002 | *-*  *F*_1, 85_ = 7.429  *F*_1, 112_ = 0.017  *F*_1, 113_ = 0.284  *F*_1, 114_ = 0.895  *F*_1, 114_ = 0.07  *F*_1, 111_ = 0.553  *F*_1, 111_ = 0.002 | -  0.140  1.000  1.000  1.000  1.000  1.000  1.000 |
|  |  |  |  |  |  |  |
|  | PC7  (3.2 %) | (Intercept)  Competition  Fork length  Sex  Competition ×  Fork length  Competition ×  Sex  Fork length ×  Sex  Competition ×  Fork length ×  Sex | 0.0181  -0.0197  -0.0001  -0.0298  0.0001  0.0199  0.0002  -0.0002 | 0.0086  0.0108  0.0001  0.0121  0.0001  0.0164  0.0001  0.0001 | *-*  *F*_1, 114_ = 2.308  *F*_1, 110_ = 0.000  *F*_1, 111_ = 6.748  *F*_1, 111_ = 1.03  *F*_1, 112_ = 0.028  *F*_1, 110_ = 3.196  *F*_1, 110_ = 1.463 | -  1.000  1.000  0.192  1.000  1.000  1.000  1.000 |
|  |  |  |  |  |  |  |
|  | PC8  (2.2 %) | (Intercept)  Competition  Fork length  Sex  Competition ×  Fork length  Competition ×  Sex  Fork length ×  Sex  Competition ×  Fork length ×  Sex | -0.0118  0.0161  0.0001  -0.0044  -0.0001  0.0058  3.12E-05  -4.72E-05 | 0.0074  0.0098  0.0001  0.0112  0.0001  0.0152  0.0001  0.0001 | *-*  *F*_1, 72_ = 0.362  *F*_1, 112_ = 0.958  *F*_1, 113_ = 0.085  *F*_1, 114_ = 6.978  *F*_1, 113_ = 0.002  *F*_1, 111_ = 0.012  *F*_1, 111_ = 0.143 | -  1.000  1.000  1.000  0.169  1.000  1.000  1.000 |
|  |  |  |  |  |  |  |
|  | PC9  (1.4 %) | (Intercept)  Competition  Fork length  Sex  Competition ×  Fork length  Competition ×  Sex  Fork length ×  Sex  Competition ×  Fork length ×  Sex | -0.0001  0.0016  2.07E-05  0.0096  -2.47E-05  0.0002  -0.0001  8.81E-06 | 0.0058  0.0077  4.59E-05  0.0088  0.0001  0.0119  0.0001  0.0001 | *-*  *F*_1, 72_ = 0.22  *F*_1, 112_ = 2.511  *F*_1, 113_ = 6.912  *F*_1, 114_ = 0.185  *F*_1, 113_ = 0.195  *F*_1, 111_ = 5.322  *F*_1, 111_ = 0.008 | -  1.000  1.000  0.175  1.000  1.000  0.412  1.000 |
|  |  |  |  |  |  |  |
|  | PC10  (1.0 %) | (Intercept)  Competition  Fork length  Sex  Competition ×  Fork length  Competition ×  Sex  Fork length ×  Sex  Competition ×  Fork length ×  Sex | 0.0068  -0.0063  -0.0001  -0.0112  0.0001  0.0131  0.0001  -0.0001 | 0.0049  0.0065  3.89E-05  0.0075  0.0001  0.0101  0.0001  0.0001 | *-*  *F*_1, 72_ = 0.921  *F*_1, 112_ = 0.332  *F*_1, 113_ = 1.816  *F*_1, 114_ = 0.216  *F*_1, 113_ = 0.308  *F*_1, 111_ = 0.278  *F*_1, 111_ = 2.138 | -  1.000  1.000  1.000  1.000  1.000  1.000  1.000 |
|  |  |  |  |  |  |  |
|  | PC11  (0.7 %) | (Intercept)  Competition  Fork length  Sex  Competition ×  Fork length  Competition ×  Sex  Fork length ×  Sex  Competition ×  Fork length ×  Sex | -0.0031  0.003  1.02E-05  -0.0085  5.46E-06  0.0133  0.0001  -0.0001 | 0.004  0.0053  3.11E-05  0.0059  4.22E-05  0.0081  4.77E-05  0.0001 | *-*  *F*_1, 91_ = 11.037  *F*_1, 112_ = 1.342  *F*_1, 113_ = 0.015  *F*_1, 113_ = 1.554  *F*_1, 114_ = 0.042  *F*_1, 111_ = 0.159  *F*_1, 111_ = 3.036 | -  0.023  1.000  1.000  1.000  1.000  1.000  1.000 |
|  |  |  |  |  |  |  |
|  | PC12  (0.7 %) | (Intercept)  Competition  Fork length  Sex  Competition ×  Fork length  Competition ×  Sex  Fork length ×  Sex  Competition ×  Fork length ×  Sex | 0.0086  -0.0155  -0.0001  -0.011  0.0001  0.0164  0.0001  -0.0002 | 0.0039  0.0051  3.05E-05  0.0058  4.14E-05  0.0079  4.69E-05  0.0001 | *-*  *F*_1, 74_ = 0.981  *F*_1, 112_ = 0.151  *F*_1, 113_ = 5.179  *F*_1, 114_ = 4.247  *F*_1, 114_ = 0.985  *F*_1, 111_ = 1.285  *F*_1, 111_ = 5.609 | -  1.000  1.000  0.445  0.749  1.000  1.000  0.353 |
|  |  |  |  |  |  |  |
|  | PC13  (0.4 %) | (Intercept)  Competition  Fork length  Sex  Competition ×  Fork length  Competition ×  Sex  Fork length ×  Sex  Competition ×  Fork length ×  Sex | 0.0001  -0.0001  -3.11E-06  -4.98E-05  4.94E-06  0.0013  3.02E-06  -1.41E-05 | 0.0033  0.0044  2.59E-05  0.005  3.53E-05  0.0067  4.00E-05  0.0001 | *-*  *F*_1, 72_ = 0.104  *F*_1, 112_ = 0.026  *F*_1, 113_ = 0.037  *F*_1, 114_ = 0.001  *F*_1, 113_ = 0.049  *F*_1, 111_ = 0.024  *F*_1, 111_ = 0.065 | -  1.000  1.000  1.000  1.000  1.000  1.000  1.000 |
|  |  |  |  |  |  |  |
|  | PC14  (0.3 %) | (Intercept)  Competition  Fork length  Sex  Competition ×  Fork length  Competition ×  Sex  Fork length ×  Sex  Competition ×  Fork length ×  Sex | -0.0028  0.0054  2.43E-05  0.0032  -4.68E-05  -0.0046  -3.18E-05  4.56E-05 | 0.0027  0.0035  2.11E-05  0.004  2.86E-05  0.0055  3.24E-05  4.50E-05 | *-*  *F*_1, 72_ = 0.084  *F*_1, 112_ = 0.134  *F*_1, 113_ = 0.171  *F*_1, 114_ = 1.589  *F*_1, 113_ = 0.406  *F*_1, 111_ = 0.13  *F*_1, 111_ = 1.026 | -  0.773  0.715  0.680  0.210  0.526  0.719  0.313 |
|  |  |  |  |  |  |  |
|  | PC15  (0.2 %) | (Intercept)  Competition  Fork length  Sex  Competition ×  Fork length  Competition ×  Sex  Fork length ×  Sex  Competition ×  Fork length ×  Sex | -0.0002  0.0003  -4.06E-06  0.0026  6.40E-06  -0.0048  -1.45E-05  3.46E-05 | 0.0022  0.0029  1.70E-05  0.0032  2.31E-05  0.0044  2.61E-05  3.62E-05 | *-*  *F*_1, 83_ = 1.361  *F*_1, 112_ = 0.005  *F*_1, 113_ = 0.697  *F*_1, 114_ = 1.269  *F*_1, 114_ = 0.437  *F*_1, 111_ = 0.035  *F*_1, 111_ = 0.911 | -  1.000  1.000  1.000  1.000  1.000  1.000  1.000 |
|  |  |  |  |  |  |  |
|  | PC16  (0.1 %) | (Intercept)  Competition  Fork length  Sex  Competition ×  Fork length  Competition ×  Sex  Fork length ×  Sex  Competition ×  Fork length ×  Sex | 0.0028  -0.0038  -1.93E-05  -0.0034  2.75E-05  0.0042  1.97E-05  -2.65E-05 | 0.0014  0.0019  1.12E-05  0.0021  1.52E-05  0.0029  1.72E-05  2.38E-05 | *-*  *F*_1, 85_ = 0.022  *F*_1, 112_ = 0.131  *F*_1, 113_ = 2.122  *F*_1, 113_ = 1.989  *F*_1, 114_ = 2.199  *F*_1, 111_ = 0.249  *F*_1, 111_ = 1.231 | -  1.000  1.000  1.000  1.000  1.000  1.000  1.000 |
|  |  |  |  |  |  |  |
|  | PC17  (0.1 %) | (Intercept)  Competition  Fork length  Sex  Competition ×  Fork length  Competition ×  Sex  Fork length ×  Sex  Competition ×  Fork length ×  Sex | -0.0013  0.0023  1.06E-05  0.0023  -1.75E-05  -0.0061  -1.77E-05  4.58E-05 | 0.0012  0.0016  9.78E-06  0.0019  1.33E-05  0.0025  1.51E-05  2.09E-05 | *-*  *F*_1, 72_ = 0.072  *F*_1, 112_ = 0.492  *F*_1, 113_ = 0.43  *F*_1, 114_ = 0.009  *F*_1, 113_ = 1.111  *F*_1, 111_ = 0.329  *F*_1, 111_ = 4.777 | -  1.000  1.000  1.000  1.000  1.000  1.000  0.557 |
|  |  |  |  |  |  |  |
|  | PC18  (0.03 %) | (Intercept)  Competition  Fork length  Sex  Competition ×  Fork length  Competition ×  Sex  Fork length ×  Sex  Competition ×  Fork length ×  Sex | -0.0002  0.0007  2.28E-06  -0.0004  -7.79E-06  0.0002  4.59E-06  -1.82E-06 | 0.0009  0.0012  7.00E-06  0.0013  9.52E-06  0.0018  1.08E-05  1.50E-05 | *-*  *F*_1, 72_ = 1.103  *F*_1, 112_ = 0.011  *F*_1, 113_ = 0.618  *F*_1, 114_ = 1.293  *F*_1, 113_ = 0.000  *F*_1, 111_ = 0.238  *F*_1, 111_ = 0.015 | -  1.000  1.000  1.000  1.000  1.000  1.000  1.000 |

.

**Table S4** Statistical results from GLMMs examining the effects of fork length (continuous), species category (white-spotted charr [WSC], allopatric southern Asian Dolly Varden [SADV], sympatric SADV, and masu salmon [MS]), and their interaction on PC1 from generalized Procrustes analysis of summer lateral body morphology in three salmonids in the sympatric reach and SADV in the allopatric reach. Coefficients are presented with WSC as the reference category

|  | Response variable | Explanatory variables | Coefficient | SE | Statistics | p-value |
| --- | --- | --- | --- | --- | --- | --- |
| Summer lateral body morphology (allopatric SADV, sympatric SADV, MS, WSC) | PC1  (47.8 %) | (Intercept)  Fork length  Species category  (WSC – sympatric SADV)  (WSC – MS)  (WSC – allopatric SADV)  Fork length × Species category  (WSC – sympatric SADV)  (WSC – MS)  (WSC – allopatric SADV) | -8.618e-03  5.801e-06  2.613e-02  -3.334e-02  4.204e-02  2.186e-05  5.661e-05  -1.597e-04 | 6.814E-03  5.094E-05  8.271E-03  8.746E-03  9.092E-03  6.220E-05  7.288E-05  7.040E-05 | *-*  *F*_1, 174_ = 0.109  *F*_3, 173_ = 310.301  *F*_3, 172_ = 3.945 | -  0.7413  < 0.001  0.009 |


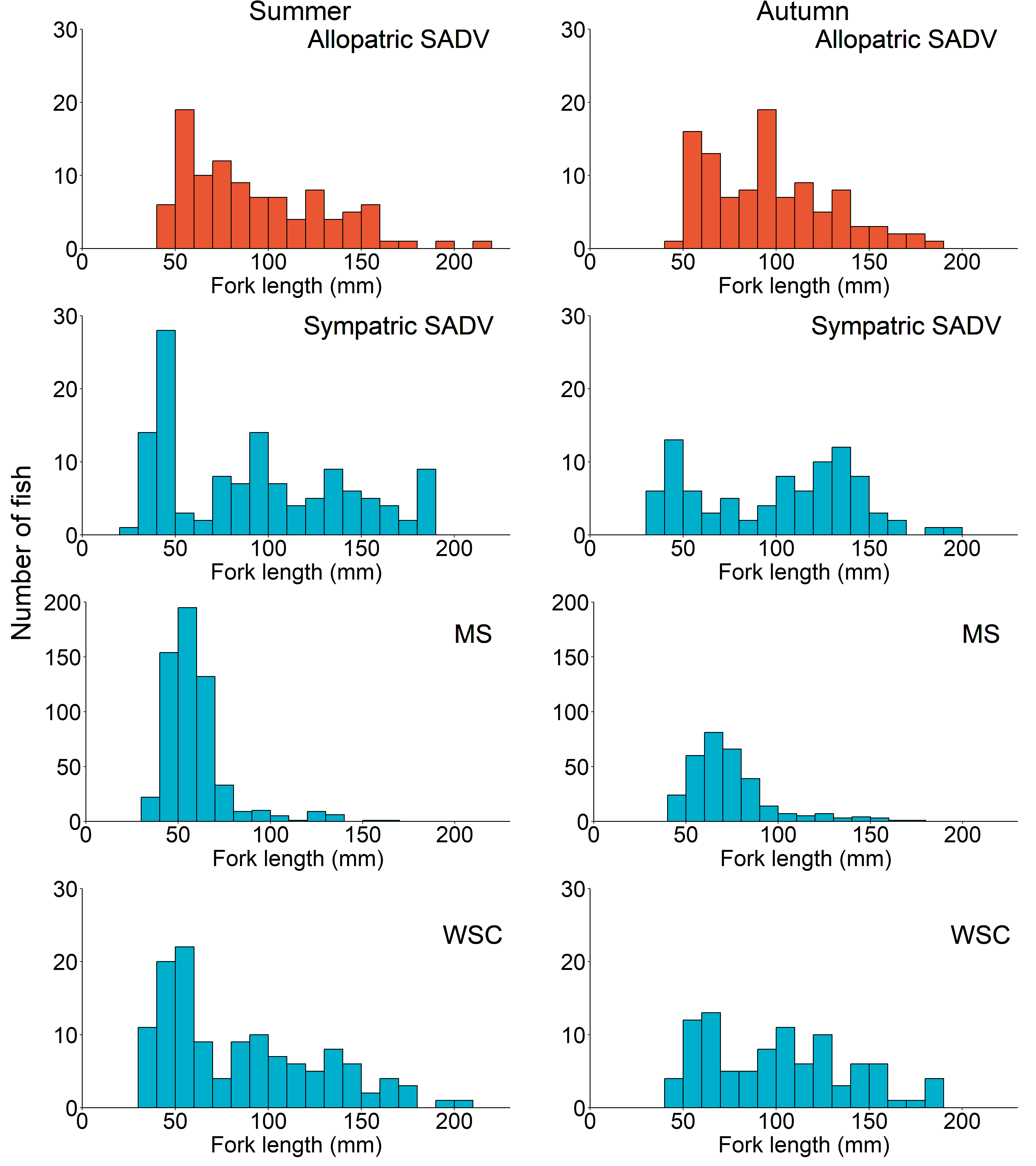


**Fig. S1** Fork length distributions of southern Asian Dolly Varden (SADV; summer, *n* = 128; autumn, *n* = 90), masu salmon (MS; summer, *n* = 735; autumn, *n* = 315), and white-spotted charr (WSC; summer, *n* = 128; autumn, *n* = 95) in the sympatric reaches and those of SADV in the allopatric reaches (summer, *n* = 101; autumn, *n* = 104) of the Setose River, Hokkaido, Japan


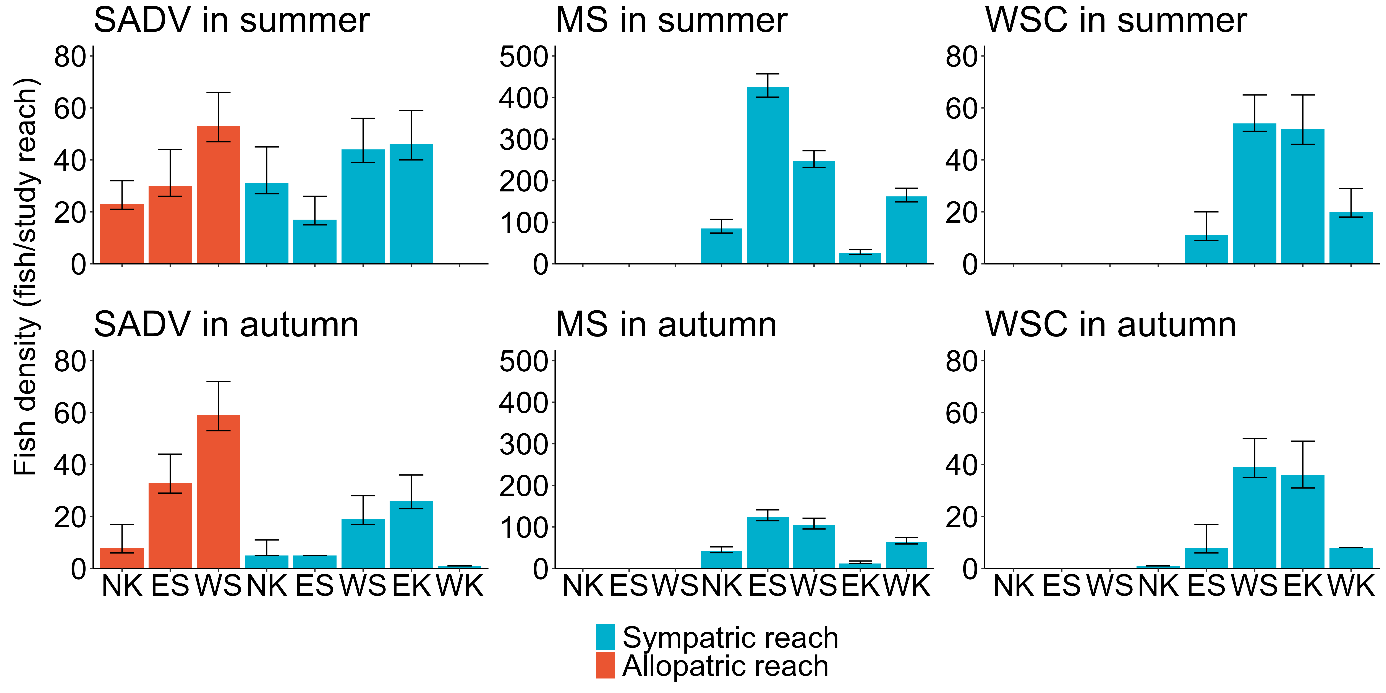


**Fig. S2** Estimated numbers (±95%CI) of southern Asian Dolly Varden (SADV), masu salmon (MS), and white-spotted charr (WSC) in each 50-m study reach. The upper bar plot represents summer data, while the lower bar plot indicates autumn data. The two-letter abbreviations refer to river names: NK for Nakasawa River, ES for East Setose River, WS for West Setose River, EK for East Kozan River, and WK for West Kozan River


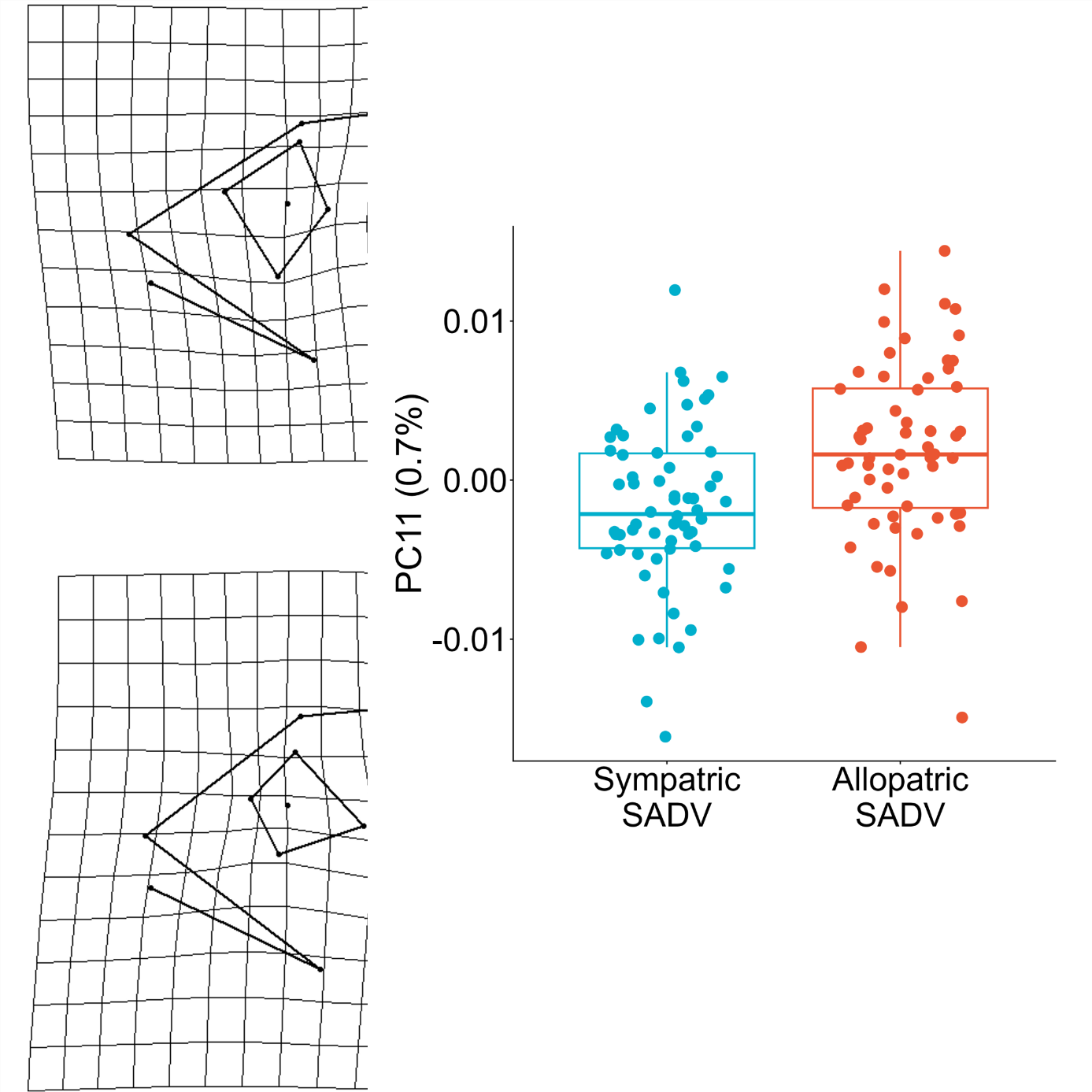


**Fig. S3** Enlarged view of the wireframe plot of head morphology for southern Asian Dolly Varden (SADV) in autumn (Fig. 5c), provided to improve visualization. Boxplot of PC11 scores derived from head morphology of SADV in autumn for sympatric and allopatric reaches. Thin-plate spline grids and wireframes, based on 11 landmarks, illustrate morphological changes along PC11 from negative to positive ends. Points in the boxplot represent individual PC scores
